# Genome-wide copy number variation association study in anorexia nervosa

**DOI:** 10.1038/s41380-024-02811-2

**Published:** 2024-11-12

**Authors:** Alicia Walker, Robert Karlsson, Jin P. Szatkiewicz, Laura M. Thornton, Zeynep Yilmaz, Virpi M. Leppä, Androula Savva, Tian Lin, Julia Sidorenko, Allan McRae, George Kirov, Helena L. Davies, Bengt T. Fundín, Samuel J. R. A. Chawner, Jie Song, Stina Borg, Jia Wen, Hunna J. Watson, Melissa A. Munn-Chernoff, Jessica H. Baker, Scott Gordon, Wade H. Berrettini, Harry Brandt, Steven Crawford, Katherine A. Halmi, Allan S. Kaplan, Walter H. Kaye, James Mitchell, Michael Strober, D. Blake Woodside, Nancy L. Pedersen, Richard Parker, Jennifer Jordan, Martin A. Kennedy, Andreas Birgegård, Mikael Landén, Nicholas G. Martin, Patrick F. Sullivan, Cynthia M. Bulik, Naomi R. Wray

**Affiliations:** 1 Institute for Molecular Bioscience, University of Queensland, Brisbane, QLD Australia; 2 Department of Medical Epidemiology and Biostatistics, Karolinska Institutet, Stockholm, Sweden; 3 Department of Genetics, University of North Carolina at Chapel Hill, Chapel Hill, NC USA; 4 Department of Psychiatry, University of North Carolina at Chapel Hill, Chapel Hill, NC USA; 5 National Centre for Register-based Research, Aarhus University, Aarhus, Denmark; 6 Department of Biomedicine, Aarhus University, Aarhus, Denmark; 7 Department of Clinical Neuroscience, Karolinska Institutet, Stockholm, Sweden; 8 Centre for Neuropsychiatric Genetics and Genomics, Cardiff University, Cardiff, Wales UK; 9 Social, Genetic and Developmental Psychiatry Centre, King’s College London, London, UK; 10 Centre for Eating and feeding Disorders Research, Mental Health Centre Ballerup, Copenhagen, Denmark; 11 Institute of Biological Psychiatry, Mental Health Centre Sct. Hans, Roskilde, Denmark; 12 School of Psychology, Curtin University, Perth, WA Australia; 13 School of Paediatrics and Child Health, University of Western Australia, Perth, WA Australia; 14 Department of Community, Family, and Addiction Sciences, Texas Tech University, Lubbock, TX USA; 15 Equip Health Inc., Carlsbad, CA USA; 16 Genetic Epidemiology Laboratory, QIMR Berghofer Medical Research Institute, Brisbane, QLD Australia; 17 Department of Psychiatry, Center for Neurobiology and Behavior, University of Pennsylvania Perelman School of Medicine, Philadelphia, PA USA; 18 The Center for Eating Disorders at Sheppard Pratt, Baltimore, MD USA; 19 Department of Psychiatry, Weill Cornell Medical College, New York, NY USA; 20 Centre for Addiction and Mental Health, Toronto, ON Canada; 21 Institute of Medical Science, University of Toronto, Toronto, ON Canada; 22 Department of Psychiatry, University of Toronto, Toronto, ON Canada; 23 Department of Psychiatry, University of California San Diego, La Jolla, CA USA; 24 Department of Psychiatry and Behavioral Science, University of North Dakota School of Medicine and Health Sciences, Fargo, ND USA; 25 Department of Psychiatry and Biobehavioral Science, Semel Institute for Neuroscience and Human Behavior, University of California Los Angeles, Los Angeles, CA USA; 26 David Geffen School of Medicine, University of California Los Angeles, Los Angeles, CA USA; 27 Centre for Mental Health, University Health Network, Toronto, ON Canada; 28 Program for Eating Disorders, University Health Network, Toronto, ON Canada; 29 QIMR Berghofer Medical Research Institute, Brisbane, QLD Australia; 30 Department of Psychological Medicine, University of Otago, Christchurch, New Zealand; 31 Canterbury District Health Board, Christchurch, New Zealand; 32 Department of Pathology and Biomedical Science, University of Otago, Christchurch, New Zealand; 33 Department of Psychiatry and Neurochemistry, The Sahlgrenska Academy at the University of Gothenburg, Gothenburg, Sweden; 34 Department of Nutrition, University of North Carolina at Chapel Hill, Chapel Hill, NC USA; 35 Department of Psychiatry, University of Oxford, Oxford, UK

**Keywords:** Genetics, Psychology

## Abstract

This study represents the first large-scale investigation of rare (<1% population frequency) copy number variants (CNVs) in anorexia nervosa (AN). Large, rare CNVs are reported to be causally associated with anthropometric traits, neurodevelopmental disorders, and schizophrenia, yet their role in the genetic basis of AN is unclear. Using genome-wide association study (GWAS) array data from the Anorexia Nervosa Genetics Initiative (ANGI), which included 7414 AN case and 5044 controls, we investigated the association of 67 well-established syndromic CNVs and 178 pleiotropic disease-risk dosage-sensitive CNVs with AN. To identify novel CNV regions (CNVRs) that increase the risk of AN, we conducted genome-wide association studies with a focus on rare CNV-breakpoints (CNV-GWAS). We found no net enrichment of rare CNVs, either deletions or duplications, in AN, and none of the well-established syndromic or pleiotropic CNVs had a significant association with AN status. However, the CNV-GWAS found 21 nominally associated CNVRs that contribute to AN risk, covering protein-coding genes implicated in synaptic function, metabolic/mitochondrial factors, and lipid characteristics, like the *CD36* (7q21.11) gene, which transports long-chain fatty acids into cells. CNVRs intersecting genes previously related to neurodevelopmental traits include deletions of *NRXN1* intron 5 (2p16.3), *IMMP2L* (7q31.1), and *PTPRD* (9p23). Overall, given that our study is well powered to detect the CNV burden level reported for schizophrenia, we can conclude that rare CNVs have a limited role in the etiology of AN, as reported for bipolar disorder. Our nominal associations for the 21 discovered CNVRs are consistent with AN being a metabo-psychiatric trait, as demonstrated by the common genetic architecture of AN, and we provide association results to allow for replication in future research.

## Introduction

Anorexia nervosa (AN) is a severe, metabo-psychiatric disorder [1] with one of the highest mortality rates among mental illnesses [2]. Characterized by low body mass index (BMI), a fear of weight gain, and restrictive eating [3], AN has a substantial genetic component, with twin-based heritability estimates of 48–74% [4]. A genome-wide association study (GWAS) identified eight single nucleotide polymorphisms (SNPs) significantly associated with AN risk, and estimated SNP-based heritability to be 11–17% (s.e. = 1.0%) [1]. Although copy number variants (CNVs) are reported to contribute to the etiologies of schizophrenia, neurodevelopmental disorders, and anthropometric traits [5–9], no prior reports have used a large, well-powered sample set to evaluate the genetic liability conferred by CNVs in AN [10–12].

CNVs are structural changes in the genome involving duplications or deletions of DNA regions ≥50 base-pairs that deviate from the normal chromosomal complement of that region [13]. CNVs are important drivers of evolution [14], operating through various mechanisms such as gene dosage effects or disruption of cis-regulatory elements [15, 16]. The DECIPHER database catalogs established genomic syndromic loci associated with diagnoses in early childhood, particularly intellectual disability (ID), autism spectrum disorder, and developmental delay [6, 7]. Nine of these syndromic CNVs [5, 17, 18] and a global enrichment of rare CNVs (odds ratio (OR) 1.12) have been reported to be significantly associated with schizophrenia [5], and the genetic correlation between schizophrenia and AN estimated from GWAS data is *r*_*g*_ = 0.19 (s.e. = 0.04) [19], hence it is relevant to test if CNVs associated with schizophrenia are also associated with AN.

CNVs can be identified from SNP array data that form the basis of most GWAS albeit with a risk of potential false positives especially for smaller events [20]. Intersection of CNVs with evolutionarily constrained elements, regulatory regions, open chromatin, and coding sequences have been proposed to prioritize CNVs for their functional importance [16]. In fact, the burden of CNVs for schizophrenia, as measured by mammalian constrained base proportion, has shown a stronger link to case-control status than CNV length or count [16]. Using a functional annotation approach, Collins et al. [15]. identified 178 disease-relevant pleiotropic dosage-sensitive loci [15] (which overlap partially with the DECIPHER syndromic loci) from a meta-analysis of nearly 1 million people.

Given that AN involves low BMI and often co-occurs with psychiatric conditions like major depression and anxiety disorders, CNVs associated with psychiatric traits and BMI are of particular interest. Many psychiatric-risk CNVs confer significant variation in BMI, with a study in the UK Biobank (UKB) highlighting that out of 54 CNVs implicated as pathogenic, 12 significantly increase BMI and 3 significantly decrease BMI [8]. The ~600 kb 16p11.2 BP4-BP5 deletion of 28 genes, and the smaller distal ~220 kb 16p11.2 BP2-BP3 deletion are two examples of well-established CNVs associated with both severe, early-onset obesity [21, 22] and psychiatric traits, such as schizophrenia [5]. Preliminary studies suggest reciprocal CNVs within the 16p11.2 region induce a “mirror” BMI phenotype [21, 23], whereby a mirror phenotypic association refers to deletions and duplications having opposite effects on the phenotype. In this example, the effect sizes estimated in a CNV-GWAS of 191 K Europeans were β_*200kb-del*_ = +5.16 kg/m^2^, β_*200kb-dup*_ = −2.08 kg/m^2^, β_*600kb-del*_ = +6.15 kg/m^2^, β_*600kb-dup*_ = −1.81 kg/m^2^[24]).

Here, we investigated the impact of rare CNVs (rCNVs) on AN risk using data from the Anorexia Nervosa Genetics Initiative (ANGI) of 7414 AN cases and 5044 controls. We tested association at 67 CNVs which are DECIPHER syndromic loci or previously reported to be associated with schizophrenia and 178 disease-relevant pleiotropic dosage-sensitive CNV regions (CNVRs), with emphasis on mirror phenotype hypothesis-driven CNVs. As a secondary analysis we conducted genome-wide rare CNV-breakpoint GWASs.

## Methods

### CNV data

CNV analyses were conducted on data from the ANGI project, an initiative of the Klarman Family Foundation. ANGI is a multi-country collaboration that aims to identify the genetic causes of AN [1, 25]. All cases from the ANGI cohort included in this study met the criteria for AN based-on DSM-IV-TR, while the controls were screened negative for a history of eating disorders [1, 25]. Detailed information on recruitment, consent, phenotyping, DNA collection, and genotyping are available in other publications [1, 25]. The study was conducted under human research ethics approvals of the University of North Carolina at Chapel Hill (13-0081), Karolinska Institute (2013/112-31/2), Southern Health and Disability Ethics Committee of the New Zealand Ministry of Health (14/STH/115), QIMR Berghofer Human Research Ethics Committee, and the University of Queensland (HE002938). CNVs were called from Illumina Global Screening Array raw intensity data (618,540 probes) from 13,787 ANGI individuals. These individuals were divided into two groups: ANGI-SWE (Sweden, 3 batches) and ANGI-ANZUS (Australia, New Zealand, USA, 3 batches). CNVs were called for autosomes and the X chromosome using EnsembleCNV [26], a software wrapper for three CNV calling algorithms: iPattern [27], PennCNV [28] and QuantiSNP [29]. We retained CNVs that were detected by at least 2 algorithms, were longer than 20 kb, included at least 10 probes, and that passed a series of quality control steps (Supplementary Materials, Supplementary Table S1*,* Supplementary Figs. S1–3). CNV analysis of ANGI was conducted on the Vector server of the Karolinska Institute, Sweden.

### Statistical analyses

We used SAIGE-GENE+ [30], PLINK (v1.7, 1.9, 2) [31], and R for analysis. Split by CNV type (i.e., deletion/duplication), we tested mean CNV count differences across ANGI-SWE batches and ANGI-ANZUS using non-parametric ANOVA. ANGI-SWE/ANGI-ANZUS membership was included as a covariate to compare rCNV burden in AN cases and controls within ANGI. The first ancestry PC showed a nominally significant association (*P* = 0.021) to small CNV burden (autosomal <100 kb CNV burden) and was included as a covariate. BMI was omitted as a covariate due to genetic association with AN (*r*_*g*_ = −0.22, *P* = 2 × 10^−4^) [19] and was similarly omitted in the recent AN GWAS [1].

### Genome-wide CNV burden

We tested whether genome-wide rCNV burden was greater in AN cases than in controls, measured as total distance, total CNV count, number of deletions intersecting haplo-insufficient autosomal protein-coding genes (pHaplo ≥ 0.86, N = 2964 genes) [15] by at least 1 base-pair (bp), number of duplications intersecting triplo-sensitive autosomal protein-coding genes (pTriplo ≥0.94, *N* = 1244 genes) [15] by at least 1bp, and the average proportion of bases within CNVs that are evolutionary constrained. PhyloP (range: −20 to 9.28) gauges constraint across 240 mammals, while PhastConst (range: 0–1) measures constraint across 43 primates [32]. Highly constrained bases are defined by PhyloP ≥2.27 (5% FDR, 3.26% human genome) or PhastConst ≥0.96 (3.26% human genome) [16]. The genome build was hg38 (GRCh38). We further measured rCNV burden partitioned across CNV types, CNV lengths, and CNV counts between AN cases and controls. Both total distance (kb) and genes affected by CNVs were highly significantly associated with schizophrenia [5] (OR 1.12 and 1.21 respectively). In schizophrenia, the mean number of genes affected per rare CNV was estimated as 2.2 in controls with an OR of 1.21 for cases. Assuming the mean number of genes per CNV is Poisson distributed, the effect size difference is ~0.3 standard deviation (SD) units. Our ANGI study has 100% power to detect the burden increase in CNVs associated with schizophrenia, and >80% power to detect a difference of 0.05 SD at a significance threshold of 0.05.

### Locus-wide rCNV associations

#### Syndromic CNVs

We compiled 67 syndromic CNVs, including 45 deletions and 22 duplications, to test for association with AN. Of these CNVs, 60 were extracted from the DECIPHER database (v11.19) [7], and nine of these 60 (e.g. 22q11.2 deletions) are associated with elevated schizophrenia risk [5, 17, 18], cognitive impairment [33], and ID [34]. Six schizophrenia-associated CNVs [5, 17, 18], such as *NRXN1* (ENSG00000179915) exonic deletion, not recognized as syndromic by DECIPHER, were also included. Some of the DECIPHER CNVs, including the 22q11.2 deletion, are associated with obesity [8, 21, 22]. To investigate whether their reciprocal counterparts induce a “mirrored” trait (i.e., AN as a lowered BMI is a primary feature of AN), we added the ~220kb 16p11.2 duplication, noting that the 22q11.2 duplication and the ~600kb 16p11.2 duplication were already part of the DECIPHER list.

#### Pleiotropic dosage-sensitive CNVs

As AN is a multi-faceted disorder [1], we also tested a CNV list of 178 dosage-sensitive genomic segments (77 deletions and 101 duplications) [15] that confer disease-risk across 54 complex and Mendelian traits/disorders, including 24 neurodevelopmental traits. Most DECIPHER CNVs are on this list, but since the boundaries were not always consistent, we retained the full set of 178 allowing comparisons with other studies that investigate this set.

#### CNV annotation and association tests

In line with prior studies [5, 10], we created general CNV annotation rules; a called CNV was annotated to the 67 developmental-associated regions if the CNV overlaps the region by 50% or that at least 50% of the CNV overlaps the region. For *NRXN1*, as in another study [33], we required overlap with an exon from the *NRXN1* transcript ENST00000401669 by at least 1bp. For the 178 disease-risk segments, we required that either the CNV is 100kb in length and that at least 50% of the CNV overlaps the segment (i.e., minimum 50kb overlap) or that the CNV overlaps the segment by 25% (i.e., minimum 50kb overlap as the smallest segment is 200 kb). Firth logistic regression model (R::logistf, appropriate because of the low number observed events at each CNV) was used for each CNV that had at least one observation (independent variable) to test the association with AN status. Significance was declared at the Bonferroni corrected level (0.05/*n*) with *n* = 67 developmental-associated CNVs (using two-sided *P*-values because direction of effect can be disease/trait dependent for some CNVs), and *n* = 178 disease-risk CNVs (using one-sided *P*-values as the authors supplied this set as disease-risk associated). As a supplementary analysis, the 67 developmental-associated loci were further assessed for their association with lowest BMI using linear regression models, within AN cases and separately within AN controls.

### rCNV breakpoint Genome-wide Association Study (GWAS)

We conducted CNV-type specific GWASs of rCNV breakpoints (each unique start/end position of a called CNV). First, CNV coordinates recorded within PLINK CNV files were transformed into two-sets of sample * probe matrices; entries reflecting CNV breakpoint encodings according to a duplication-only model or a deletion-only model. For the duplication-only model, the following encodings were used: copy neutral as AA, duplications as AT, and deletions as 00 (i.e., as missing). For the deletion-only model, the reciprocal encodings for deletions and duplications were used. Any CNV overlapping a unique breakpoint was counted as an observation for the breakpoint. Both models did not consider extra dosage effects of the loss or gain of more than one copy. Finally, the matrices were converted into PLINK binary files and alternate (T) allele counts for CNV breakpoint-probes were extracted (PLINK2 --geno-counts) and converted into frequencies. SAIGE-GENE+ software [30] was used for CNV breakpoint GWAS under duplication-only or deletion-only models. Restricting to rare breakpoints, CNV region (CNVR) were defined by the most distant breakpoint with a r^2^ ≥ 0.5 within 300 kb of each lead, independent and nominally significant (*p* ≤ 0.05) rare breakpoint. Finally, we required that CNVRs are ≥20 kb in length, span at least 10 probes, with AN risk effect. Identified CNVRs were annotated with measures of evolutionary constraint (PhyloP and PhastConst), for which we report the maximum score for each CNVR, and the proportion of highly constrained bases [16].

### Replication study

Given that the key interest is rare CNVs, the UKB was considered to maximize sample size in addition to the ANGI cohort. However, while ANGI participants were recruited with specific phenotype guidelines, the UKB case control definitions are based on electronic health record codes and self-reported diagnoses. The UKB cohort includes both AN and atypical AN (AAN) (i.e., met all criteria for AN except low weight) (*N* = 1260) and is notably older at recruitment (UKB cases ~53 years, Supplementary Table S2) than the typical age of onset of AN and hence the age at lowest recorded BMI in ANGI is much younger (ANGI cases ~16 years, Table 1). Therefore, at the cost of phenotypic heterogeneity, the likelihood of replicating rare AN-associated variants detected within ANGI in the UKB is low, impacting the validity of meta-analyses results. Moreover, the UKB and ANGI used different GWAS arrays, potentially impacting inter-dataset reproducibility. See supplementary materials for UKB methods, results, and meta-analyses results with ANGI, which were conducted at the University of Queensland.

**Table 1:** Study characteristics of the ANGI cohort.

			BMI (kg/m^2^)			Age (years)			AN PGS^a^	
AN status	Sex	*N*	Mean	SD	Missing (%)	Mean	SD	Missing (%)	Mean	SD
Control	All	5044	20.9	2.0	18	21.1	8.2	98	0	1.00
	Female	4759	20.8	1.9	19	21.6	8.6	99		
	Male	285	22.1	2.4	2	19.8	7.1	92		
Case	All	7414	15.2	2.1	41	15.6	4.4	30	0.54	1.12
	Female	7263	15.1	2.1	42	15.6	4.3	29		
	Male	151	16.3	1.9	36	17.5	4.6	38		

The ANGI cohort is analyzed as two sets ANGI-SWE (3664 cases; 3661 controls) and ANGI-ANZUS (3750 cases; 1383 controls).

*BMI* lowest body mass index, *SD* standard deviation, *PGS* polygenic scores.

^a^PGS were calculated as the weighted sum of SNP allele counts, with the SNP weights derived from standard Clumping and *P*-value Thresholding (C + PT) applied to genome-wide association study (GWAS) summary statistics from the Watson et al. study (see supplementary note for full methods). PGS were standardized within each of the two ANGI sets (ANGI-ANZUS, ANGI-SWEL) so that controls had PGS with mean zero and standard deviation of 1. Across both ANGI sets, the PGS AUC statistic was 0.588 (*P*-value: 7.4 ×10^−53^). The odds ratio for being a case in the tenth compared to the first deciles of polygenic risk was 2.66 (95 CI 2.24–3.15).

## Results

### Total rare CNV burden in AN

Following CNV calling and quality control, 37,464 CNVs from 12,458 unrelated samples (95% inferred European) were available for analysis. This included 7414 cases (98% female) and 5044 (94% female) controls. Consistency in CNV rates across batches indicated cross-batch validity (Supplementary Fig. S4). Rare (sample frequency <1%) CNVs had median size of 102.8 kb, averaging 1.6 CNVs (0.9 duplications and 0.7 deletions) per individual. Phenotype summaries, including BMI, age and AN polygenic risk score are provided in Table 1.

**Fig. 1:**
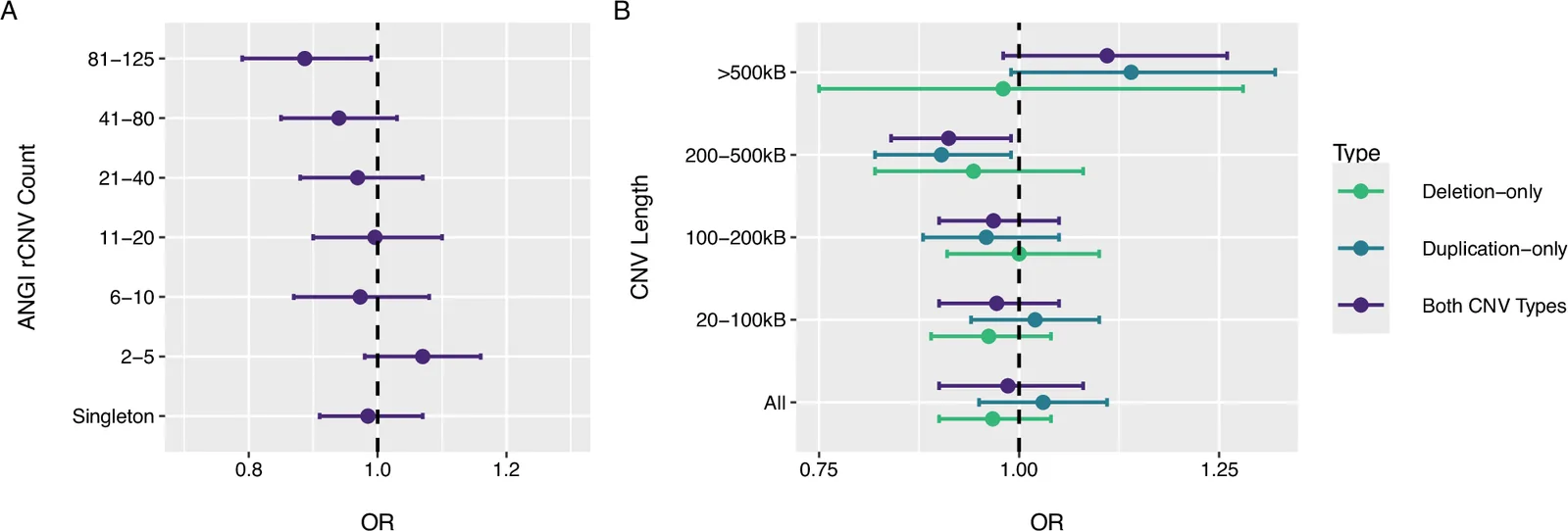
Total genome-wide rare CNV burden. **A** We tested whether genome-wide rCNV burden was greater in AN cases than in controls. **A** Partitioning the genome-wide rCNV burden by frequency (based on 50% reciprocal overlap with the full rCNV call set), no enrichment of singletons or rCNVs with counts up to 125 (1% frequency) was observed. **B** Partitioning total rCNV burden across CNV types and CNV lengths, no significant rCNV burden enrichment was observed across any length range, except for a slight excess of large (>500 kb) duplications (OR: 1.40, CI: 0.99–1.32, one-sided *P*: 0.037).

Total rCNV burden was not greater among AN cases than controls when measured as total distance covered with every 100 kb (OR: 1.00, CI: 0.99–1.01, P: 0.65) or when measured as total CNV count (OR: 0.98, CI: 0.96–1.01, *P*: 0.89) (Supplementary Table S3). Moreover, the burden of rCNVs affecting genes with dosage sensitivity [15] showed no enrichment of deletions affecting haploinsufficient genes (OR: 1.05, CI: 0.90–1.21, P: 0.28) and no enrichment of duplications affecting triplosensitive genes (OR: 1.10, CI: 0.92–1.32, P: 0.15) (Supplementary Table S3). Further analysis examining genome-wide rCNV burden across length range (20–100 kb to >500 kb) and frequency range (singletons to 1% frequency), as well as split by CNV type, did not reveal any genome-wide significant enrichment associated with AN (Fig. 1). Although evolutionary constrained base proportion had a stronger link to disease status in schizophrenia than other CNV features (e.g., CNV length) [16], we found no significant association for AN when investigating all mammalian constraint bases or primate constrained bases (but confidence intervals of estimates were wide Supplementary Table S3). Further partitioning by CNV type did not show any significant CNV burden divergence in the proportion of primate constrained base-pairs between AN cases and controls, although duplications had a larger effect size (OR: 3.32, CI: 0.34–32.76, *P*: 0.15) than deletions (OR: 0.853, CI: 0.09–8.26, *P*: 0.56) (Supplementary Table S3).

### Syndromic and pleiotropic dosage-sensitive CNV associations

Among the 67 annotated syndromic CNVs, 19 (28%) were unobserved within ANGI. Two-sided association analyses were performed for each locus with AN status (48 tests), yet none yielded significant associations with AN status (Supplementary Table S4). None of the 15 loci, previously implicated in schizophrenia and ID, including 16p11.2, *NRXN1*, 22q11.2 and 3q29 deletions, displayed significant associations with AN (Supplementary Table S4). Next, we expanded the analysis to 178 CNVRs previously reported to play a pleiotropic role in the development of 54 complex and Mendelian traits/disorders including 24 neurodevelopmental traits [15]. Among these CNVRs, 54 (30%) had no observations, and none exhibited significant AN-risk after correcting for multiple testing (Supplementary Table S5). None of the well-established 16p11.2 duplications, both distal and proximal, associated with low BMI in the UKB [24] were found to increase risk of AN. When analyzing BMI within AN cases, none of the 67 syndromic loci, including 16p11.2 duplications, showed a statistically significant association with BMI (Supplementary Table S7)*.* In fact, none of the UKB participants with a recorded AN diagnosis carried the 16p11.2 duplications known to be negatively associated with BMI. In the large UKB control set, we replicated the previously reported UKB findings of the BMI-reducing effects of 16p11.2 duplications and the BMI-increasing effects of 16p11.2 deletions (Supplementary Table S8). In ANGI controls, the directions of effect aligned with these results, but the number of CNVs (2 duplications and 2 deletions) was too small to draw strong conclusions (Supplementary Table S8). See supplementary material for full details of the CNV-BMI association results.

### Rare CNV breakpoint GWAS for novel AN-risk CNVR identification

**Fig. 2:**
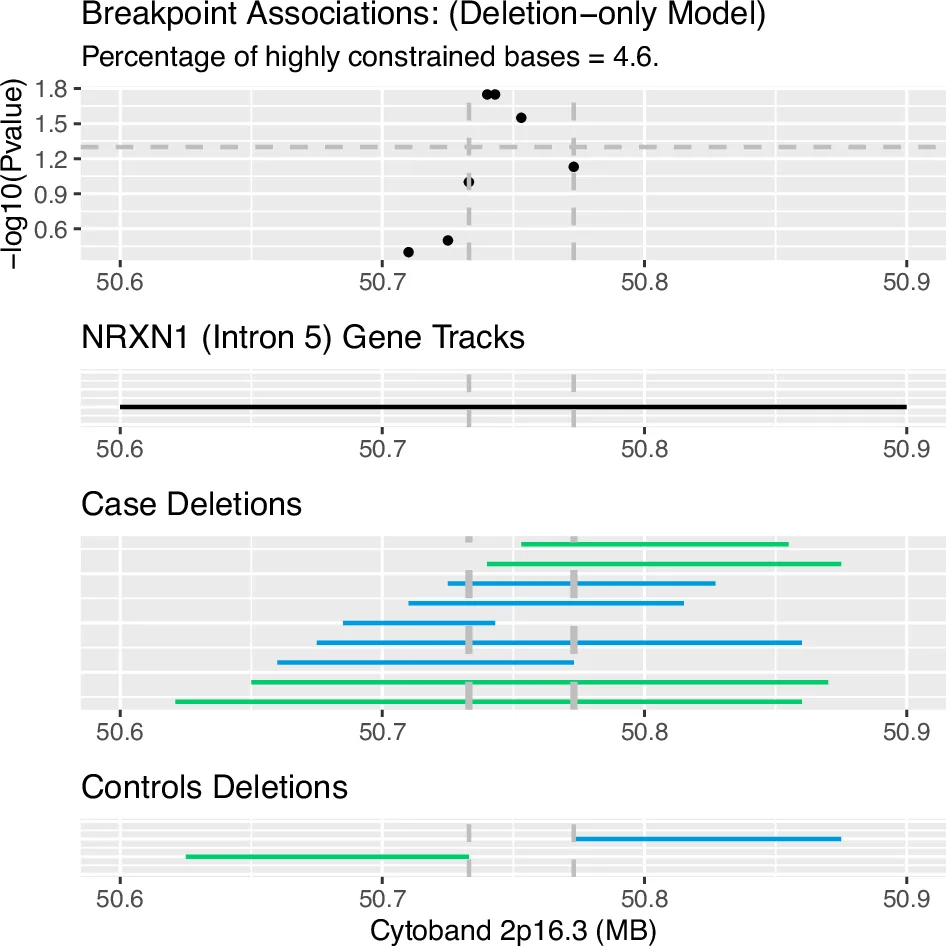
Rare CNV breakpoint association results for the ~40 kb NRXN1 intron 5 deletion. The two vertical gray dashed lines within all panels illustrate the critical region of the AN-risk CNVR. The top panel illustrates SAIGE rCNVb associations from the deletion-only model within the defined ~40 kb critical CNVR boundary and a buffer region of ±40 kb. 4.6% of the base pairs in this CNVR are annotated as highly constrained, an annotation given to 3.54% of the genome. Gene tracks panel shows the intersecting protein-coding gene tracks extracted from the UCSC genome browser (v43). The bottom two panels illustrate the tracks for CNVs found within AN cases and controls intersecting the CNVR by at least 1 base-pair, with green CNVs indicating individuals from ANGI-ANZUS, and blue CNVs indicating individuals from ANGI-SWE. The number of CNVs per cohort contributes to the differences in the test statistic and -log10 *p*-value across the x-axis.

We conducted a GWAS analysis for the copy-number of CNVs at CNV breakpoints, split by CNV-type, to facilitate the discovery of novel AN-associated loci. The analysis focused on 18,873 rare CNV breakpoints (14,296 duplications, 10,845 deletions), with 364 duplications and 213 deletions surpassing nominal significance (*P* < 0.05), though none reaching Bonferroni threshold significance. Three signals on cytoband 5p12 had nominal significance in both CNV type-specific models (Supplementary Fig. S5), with duplications offering AN protection and deletions posing AN-risk implying “mirror” effects. After defining CNVRs, 21 AN-risk CNVRs were nominally significant (two-sided *P* < 0.05) (Table 2). Ranging from 25.7 kb to 2.14 Mb (average ~295 kb), 12 (57%) of these CNVRs intersected 1-2 protein-coding genes and 7 (33%) were highly constrained (mean constraint score across base pairs greater than threshold expected for 3.26% of the genome). CNVR-specific plots illustrate SAIGE rCNV breakpoint associations and CNV overlaps, protein-coding genes, and evolutionary constraint (Fig. 2, Supplementary Figs. S6.01–6.20).

**Table 2:** AN-risk CNVR associations from CNV-type specific rare CNV breakpoint GWASs.

Length									phyloPConst proportion
Cytoband	Genotype	(kb)	Probes	Cases	Controls	OR	*P*-value	Genes	
3q12.1	Duplication	218	29	2	0	13	0.046	*CMSS1 FILIP1L*	7.8
12p12.3	Deletion	208	38	4	1	8.7	0.049	*MGST1*	6.1
15q21.3	Deletion	125	37	4	0	15	0.02	*ALDH1A2*	5.5
2p16.3	Deletion	40	17	9	2	13	0.018	*NRXN1*	4.6
6p24.3	Duplication	1534	262	5	0	11	0.037	*TFAP2A GCNT2*	4.5
13q14.11	Duplication	198	18	3	0	13	0.047	*VWA8*	3.7
17q12	Duplication	132	15	6	1	5.2	0.041		3.4
15q21.3	Duplication	134	11	24	7	2.3	0.035	*CGNL1*	3.3
16q21	Deletion	63	13	3	0	15	0.025		3.2
15q26.2	Duplication	2140	242	7	1	7	0.046	*MCTP2*	3.2
7q31.1	Deletion	100	13	10	3	7	0.046	*IMMP2L*	3.0
7q21.11	Deletion	50	15	3	0	42	0.013	*CD36*	2.4
5q34	Deletion	53	13	7	1	5.8	0.046		2
21q22.11	Duplication	50	19	15	4	4.7	0.0086		1.9
7p22.1	Duplication	388	22	6	2	8.8	0.048	*C1GALT1*	1.8
14q21.1	Deletion	129	18	6	1	11	0.038		1.4
2p22.3	Deletion	268	65	23	8	2.3	0.032		1.4
9p23	Deletion	26	11	9	1	5.9	0.013	*PTPRD*	0.99
1q31.1	Deletion	112	14	10	1	4.9	0.036		0.88
15q11.2	Deletion	137	36	65	36	1.7	0.043		0.043
5p12	Deletion	80	12	5	0	11	0.038		0.0

Listed CNVRs that are nominally associated with AN-risk (*P* < 0.05, two tailed test) and are ordered in descending phyloPConst proportion. phyloPConst proportion is the proportion of base pairs in the CNVR annotated to be constrained within primates (the genome-wide expectation is 3.26%). For full details see Supplementary Table S5.

In this rCNV breakpoint GWAS, a ~50 kb duplication on 21q22.1 (chr21:32.05–32.1 Mb) achieved the strongest association with AN (OR: 4.7, CI: 1.3–17.1, *P*: 0.0086, 15 cases vs 4 controls, Table 2)*,* however, the CNVR neither intersects a protein-coding gene nor is highly constrained (Supplementary Fig. S6.20). Trisomy 21 or large partial duplications of chromosome 21 have been shown to lead to severe clinical phenotypes, including Down syndrome, hypotonia, and cognitive deficits, however the smaller novel AN-risk CNVR does not fall within the chromosome 21 critical region suggested to be essential in producing the main Down syndrome features [35–37]. The CNVR showing the greatest risk for AN with an OR of 42 (CI: 2–804, *P*: 0.13) is a ~50 kb deletion within the gene *CD36* on cytoband 7q21.11 (chr7:80.6–80.7 Mb), with all three non-recurrent CNVs within this region being AN-exclusive, varying in size (Table 2*,* Supplementary Fig. S6.08). *CD36* is a membrane glycoprotein that facilitates the transportation of long-chain fatty acids into cells, participating in muscle lipid utilisation and adipose energy storage [38]. It has been shown that *CD36* deficiency in humans shows significantly decreased fatty acid uptake in muscle and adipose tissues [39], while in mice, that pharmacological inhibition of receptor protein *CD36* significantly reduces body weight gain and improves glucose tolerance [40].

The largest CNVR exclusive to AN cases (OR: 11, C: 1–148, *P*; 0.037) is a highly evolutionary constrained 1534 kb duplication on cytoband 6p24.3 (chr6:9–10.5 Mb), which disrupts the transcription factor AP-2 alpha gene, *TFAP2A*, and the glucosaminyl (N-acetyl) transferase 2 gene, *GCNT2* (Table 2*,* Supplementary Fig. S6.06). The potential relevance of *GCNT2* in Attention-deficit/hyperactivity disorder (ADHD) has been previously described, with *GCNT2* CNVs only being found in ADHD patients and none in controls in earlier genome-wide analyses [41, 42]. Similar to *GCNT2*, CNVs intersecting the protein tyrosine phosphatase gene, *PTPRD*, are more frequent in ADHD patient than in controls [41, 42]. Our study echoes this *PTPRD* disease-risk effect within AN, discovering a ~26 kb deletion on cytoband 9p23 (chr9:9.80–9.82 Mb) within the *PTPRD* gene significantly associated with AN (OR: 5.9, CI: 1.2–28.4, *P*: 0.013, 9 cases vs 1 controls) (Table 2*,* Supplementary Fig. S6.10). Genetic mutations within the *PTPRD* gene have been linked to various neurodevelopmental disorders, including ID [43], autism spectrum disorder [44], obsessive compulsive disorder [45], and restless leg syndrome [46]. Further related to CNVs previously implicated in neurodevelopmental disorders, we discovered a ~100 kb deletion CNVR on 7q31.1 (chr7:111.1–111.2 Mb) within the *IMMP2L* gene (Table 2, Supplementary Fig. S6.09), which regulates mitochondrial reactive oxygen species levels. Mutations in *IMMP2L* have been linked to Alzheimer’s disease [47], ID [48], and Tourette’s syndrome [49].

Lastly, a ~40 kb deletion within *NRNX1* overlapping intron 5 (chr2:50.73-50.77 Mb) was also found to have a strong risk association with AN (OR: 13, CI: 1–136, *P*: 0.018) (Table 2*,* Fig. 2). 4.6% of the base pairs within the CNVR are annotated to have high evolutionary constraint, and all nine non-recurrent deletions in this intronic CNVR were AN-exclusive, varying in size (Fig. 2). *NRXN1* deletions were tested in both the 67 syndromic CNV list (chr2:49.91–51.23 Mb, 1.1Mb deletion, and with the requirement of a 1 bp overlap with an exon, 6 cases vs 2 controls, *P* = 0.75, Supplementary Table S4), and in the 178 disease-risk CNVs (chr2:50.75–51.00 Mb, 250 kb deletion, 13 cases vs 4 controls, *P* = 0.19, Supplementary Table S5), though not reaching significance in either. Although the ~40 kb deletion is intronic and previous studies note that intronic deletions have milder impact than exons on neurodevelopmental disorders like autism spectrum disorder and schizophrenia [50, 51], 14 individuals with diverse neurodevelopmental phenotypes carrying an intron 5 deletion have been previously reported [52].

## Discussion

In this study, we investigated the relationship between rare CNVs and AN, and despite being very well-powered to detect an increased burden of rare CNVs, as observed in schizophrenia [5], our analysis did not provide evidence of a global enrichment of rare CNVs in AN. This lack of enrichment persisted even when we narrowed the burden test to CNVs covering dosage-sensitive genomic segments or to the proportion of evolutionary constrained base-pairs within CNVs.

None of the well-established syndromic or pleiotropic disease-risk CNV emerged as a significant risk factor for AN after multiple-testing correction. All 15 CNVs previously associated with schizophrenia yielded null results in AN, suggesting distinct genetic architectures from the perspective of large, rare CNVs for AN compared to schizophrenia, despite a common SNP-based estimate of genetic correlation of 0.19 (s.e. = 0.04) [19]. The absence of a global burden of rare CNVs, in line with previous observations in bipolar disorder [53, 54], and the lack of enrichment of schizophrenia-associated CNVs within AN are perhaps not surprising, given the strong evidence of the association of these CNVs with ID [34]. Unlike individuals with schizophrenia [55, 56], those with AN or those at risk of developing an eating disorder have been reported to exhibit higher intelligence quotient (IQ) levels compared to the general population [57, 58]. The latest (ANGI) GWAS for AN reported a significant SNP genetic correlation of 0.25 (s.e. = 0.03) with education attainment, although estimated genetic correlations with various measures of intelligence were not significantly different from zero [1]. Given that 16p11.2 duplications, both distal and proximal, are associated with low BMI in the UKB [24] (Supplementary Tables S7 and S8) we hypothesized that these should be associated with increased risk of AN. However, we found no such association and in fact only two ANGI AN cases had CNVs in this region, with the frequency 2/7414 = 0.03% not significantly different to the frequency in UKB controls 157/385930 = 0.04%. While these results could be consistent with a different genetic mechanisms underlying low BMI and AN, much larger data sets are needed to draw conclusions.

To identify CNVRs that could be a novel genetic risk factor for AN, we conducted CNV-type specific genome-wide association analyses with a focus on rare CNV breakpoints. The discovery of 21 nominally associated CNVRs highlights the potential role of synaptic function, metabolic/mitochondrial factors, and lipid characteristics in the rare genetic architecture of AN. Protein-coding genes that intersect these candidate disease-risk CNVRs for AN, such as *CD36*, *GCNT2*, *PTPRD*, *IMMP2L*, and *ALDH1A2* (among others listed in Table 2), supports the perspective of AN as a metabo-psychiatric disorder, which is consistent with the common genetic architecture of AN [1]. Furthermore, many of these candidate genes have been linked to a variety of neurodevelopmental disorders, demonstrating pleiotropic effects and emphasizing AN’s genetic heterogeneity. Additional research is needed to elucidate the specific role of these genes in the pathophysiology of AN.

We acknowledge some important limitations in our analyses. Despite adhering to general CNV annotation rules (overlap criteria of CNVs with the 67 syndromic CNVs and 178 pleiotropic CNVRs), the rules are rather lenient and somewhat arbitrary. Although we adopted annotation rules similar to those used in large CNV studies [5, 10], there is a potential reduction in the clinical significance of any identified associations for established syndromic CNVs, given that syndromic CNVs often encompass most of their defined critical regions [7]. Lastly, the absence of an appropriate replication study to conduct a feasible meta-analysis with ANGI limits the robustness in the validity of our conclusions. However, we provide the association results for the 21 identified novel AN-risk CNVRs for further replication in a new study with clinical diagnoses of AN.

Overall, this is the first large-scale genome-wide study investigating the contribution of CNVs to AN, providing important insights into the genetic etiology of the disease. The study, which was well-powered to detect CNV burden, indicates that CNVs do not play a significant role in AN and that unlike schizophrenia and neurodevelopmental disorders, AN is unlikely to be a referral reason for CNV testing by medical genetic services. It is important to note that these findings only relate to AN, and more research is needed to understand the role of CNVs in other eating disorders like bulimia nervosa, binge-eating disorder and avoidant/restrictive food intake disorder (ARFID).

## Supplementary information

Supplementary Table 1

Supplementary table 2

Supplementary Table 3

Supplementary Table 4

Supplementary Table 5

Supplementary Table 6

Supplementary Table 7

Supplementary Table 8

Supplementary Methods Results Figures

## Data Availability

The ANGI data availability and associated accession codes are available at https://doi.org/10.1038/s41588-019-0439-2. UKB data access policies (http://www.ukbiobank.ac.uk/register-apply/) and a description of the genetic data (http://www.ukbiobank.ac.uk/scientists-3/genetic-data/) are available from the UK Biobank website.

## Code availability

The GitHub containing the pipeline to conduct a CNV-GWAS and the utilized CNV annotation files are available at https://github.com/walkeralicia/Anorexia_CNV_GWAS.
