# Supplementary material for: Genome-wide copy number variation association study in anorexia nervosa
Source: Mol Psychiatry. 2024 Nov 12;30(5):2009–16. doi: 10.1038/s41380-024-02811-2 (PMC12014356; doi:10.1038/s41380-024-02811-2)
Supplement: Supplementary file 9 — Supplementary Methods Results Figures [file 41380_2024_2811_MOESM9_ESM.docx]

**Genome-wide copy number variant association study in anorexia nervosa**

**Supplementary Material: Methods, Results, and Figures**

**Supplementary methods: ANGI**

**Cohort description**

Main analyses were performed in data from the Anorexia Nervosa Genetics Initiative (ANGI), an initiative of the Klarman Family Foundation. ANGI aims to identify the genetic causes of anorexia nervosa (AN) and involves international research teams in the US, Sweden, Denmark, and Australia with assistance from New Zealand(1, 2). The data here are a subset of those used in the Watson et al. GWAS paper(1). Specifically, ANGI-DK (Denmark) (N=129) was not available to us and only cases from ANGI-ANZ (Australia/New Zealand) were available for analysis. Two data sets were available from the USA (ANGI-US and Price Foundation (controls only)) and two data sets from Sweden (ANGI-SWE (in two genotyping batches) and Lifegene). All cases from the ANGI cohort included in this CNV study (ANGI-ANZ, ANGI-US, and ANG-SWE) met the criteria for AN based-on DSM-IV-TR, while the controls screened negative for a history of eating disorders(1, 2). More details on participant recruitment, phenotyping and human research ethics approvals have been reported previously(1, 2). All ANGI analyses were conducted on the Vector Server hosted by the Department of Medical Epidemiology and Biostatistics at the Karolinska Institute in Sweden.

**Genotype data and pre-processing for CNV calling**

In ANGI, whole blood samples provided DNA for genotyping on Illumina Global Screening Array (618,540 probes) at the Broad Institute in 6 batches. Probe clustering on raw intensity data from 13,787 individuals across the 6 datasets was conducted in Illumina GenomeStudio using the CNVPartition plug-in. Exclusions applied to samples with LogRDev > 2 SD from mean, BAlleleDev > 2 SD from mean, < 98% genotyping rate, or large fragmented CNVs. Per-batch PLINK binary files were generated, and PLINK1 was used to exclude samples with excessive heterozygosity in autosomes (5 SD from mean inbreeding coefficient), and to calculate the sex concordance of the provided genders and the genotype derived sex. Those with a missing recorded gender were substituted with their predicted genotype sex. 12,766 individuals passed QC, entering downstream CNV calling (batch-level summaries in ***Table S1***). We did not perform QC at the probe-level (a typical QC filter for GWAS), because SNP probes with high missing genotype rates and deviation from Hardy-Weinberg equilibrium may be associated with CNVs(3). The intensity metrics for samples that passed QC were exported into sample-level GenomeStudio Report TXT files with the required columns for CNV calling; Sample ID, SNP Name, Chr, Position, B Allele Frequency (BAF), Log R Ratio (LRR), X value, Y value, Allele1 – Forward, Allele2 – Forward.

**CNV calling**

To mitigate false positives, three algorithms—iPattern(4), PennCNV v1.0.5(5) and QuantiSNP(6)—were employed for CNV calling per participant. EnsembleCNV(3) integrated the three algorithms, scripts adjusted for X chromosome calls. PennCNV and QuantiSNP use individual-wise analysis, sensitive for rare large CNVs. X-chromosomes were called separately from autosomes for PennCNV. In contrast, iPattern performs joint analysis over 100-500 individuals at a time, making it sensitive to detect more common CNVs. For iPattern, SNPs on Chr21p were removed owing to a low number of SNPs in this region. Since some of the contributing datasets are fully confounded with case-control status, we ran iPattern with the US and Australia/New Zealand batches (ANGI-ANZUS: ANGI-US, Price Foundation, ANGI-ANZ) combined and then with Swedish batches (ANGI-SWE: the two ANGI-SWE batches, Lifegene) combined, so that both cases and controls were present in each iPattern set analysis. Males were evenly distributed across each iPattern set for LRR variance normalization.

**Participant inferred ancestry**

Participant ancestry was inferred from genome-wide SNP data. Briefly, sample-level QCed batch PLINK files were combined into one dataset. SNPs with low genotyping call rate (< 0.90), outside Chr 1-23, Hardy-Weinberg equilibrium violation (P < 10-6), or minor allele frequency (MAF) < 0.01 were removed. SNPs were aligned to the Illumina build 37 Source strand corresponding to the array panel used to genotype the participants (GSA-24v1-0_A1-b37.Source.strand), and Illumina SNP Ids were updated to GRCh37.p5 IDs used in the 1000 Genomes Phase 3 reference data. SNPs that mismatched with the reference genome or were outliers in allele frequency (defined by a quadratic function applied on the standard deviation of the difference between the reference allele frequency and the batch SNP MAF). Principal component (PC) derived ancestry was determined from the first two PCs estimated from genome-wide SNP data including five reference cohorts (1000 Genomes Phase 3 EUR, AFR, EAS, SAS, AMR). Ancestry was defined by the mean and SD of PCs 1-3 in each ancestry.

**Detecting batch effects and sample outliers**

PC analysis was applied to CNV-calling per-sample summary statistics (SD of LRR, SD of BAF, wave factor in LRR, BAF drift, and the number of CNVs detected) to identify outliers or batch effects (as recommended in the EnsembleCNV workflow). In a simple linear regression model, we formally investigated the variance explained by batches on PC1-3 of the PC analysis (PCA). Ultimately, we excluded outlier samples (N=91; 48 ANGI-US, 16 Price Foundation, 16 ANGI, 5 Lifegene, 3 ANGI-SWE batch 1, 3 ANGI-SWE batch 2) where PC1, 2, or 3 was greater than 4 SD from the mean for this PCA (***Figure S1***). We further noted samples with an excessive total length, defined as total CNV length greater than 6 SD from the median within each CNV calling algorithm (***Figure S2***). We reran PCA on the sample-level summary statistics for the 12,675 individuals that passed QC and present a PC2 vs PC1 plot with each individual colored by batch, AN status, or PCA-derived ancestry (***Figure S3***).

**CNV quality control**

We concatenated the CNV calls within each of the two data sets used for CNV calling (i.e., ANGI-ANZUS and ANGI-SWE) for each of the three detection methods. We applied basic QC, removing low-confidence CNVs spanning < 10 probes, confidence score < 10 for PennCNV, Log Bayes Factor < 10 for QuantiSNP, and score <1 or complex CNVs for iPattern. For each CNV calling algorithm, we annealed adjoining CNVs that appeared to be artificially split by recursively joining CNVs if the called region is ≥ 75% of the entire region to be joined and then removed CNVs of length < 20kb. Finally, we removed CNVs with > 50% reciprocal overlap with large genomic gaps (e.g., centromeres) or regions subject to rearrangement in white blood cells. The remaining concordant CNVs were merged across CNV calling algorithms using outer probe boundaries, requiring CNVs to be identified in the same subject, same CNV type, and >50% reciprocal overlap. CNVs detected by only one of the three algorithms were excluded from further analysis. This approach allowed us to maximize specificity (detected by ≥2 algorithms) as well as sensitivity (combining all concordant calls among three complementary algorithms). Lastly, we converted the genomic start and end coordinates of all CNVs from hg19 to hg38 using the UCSC LiftOver Command Line Tool(7), requiring that 95% of the bases must remap. CNVs that failed to lift over and were dropped from further analyses.

**Sample selection**

After CNV calling, individuals (N = 22; 11 ANGI-US, 6 Lifegene, 2 ANGI-ANZ, 1 Price Foundation, 1 ANGI-SWE batch 1, 1 ANGI-SWE batch 2) whose biological sex was discordant from their genotype sex were excluded (reported men with an F-statistic < 0.8 and reported females with an F-statistic > 0.2). Within each inferred ancestry group, we used GCTA to eliminate related participants such that no pair of individuals had relationship coefficient (r) > 0.2 (N=195; 189 Europeans, 2 AMR, and 4 "others"). All analyses were conducted on 12,458 unrelated individuals of whom 95.3% are of inferred white European ancestry, 96.5% are females, and 59.5% are AN cases. 8,471 individuals had an available lowest BMI measurement, of whom 51.2% are AN cases, 95.6% are female, and 60.1% are from the ANGI-ANZUS cohort.

**Anorexia nervosa PGS**

Three sets of leave-one-out (LOO) AN GWAS summary statistics from the Watson et al.(1) study were obtained with the cohorts left out in turn being ANGI-US + Price Foundation, ANGI-ANZ and ANGI-SWE. SNPs which had a low per-SNP sample size (N<1st Decile) were excluded and SNPs shared between the LOO-summary stats file and the left-out cohort were retained. Polygenic score SNP weights were derived by Clumping and P-value Thresholding (C+PT) applied to each of the LOO data retaining SNPs with P-value threshold of 0.05, independent at LD r^2^ > 0.1. We calculated the polygenic score (PGS) of an individual in each left-out target cohort as the weighted sum of SNP allele counts, with the per allele weights for the SNPs derived from the corresponding LOO C+PT. Since the AN PGS for the target ANGI-AUNZ cohort only consisted of cases we standardized their PGS to be on the same scale as the cases within ANGI-US and combined them into a new target cohort. AN PGS were standardized within each cohort (ANGI-ANZUS, ANGI-SWE) so that the cohort controls had PGS with mean zero and standard deviation of 1.

**Supplementary methods: Replication study: the UK Biobank (UKB)**

**Cohort description**

Replication analyses was performed in the UKB, a volunteer-based cohort of around ~500,000 individuals aged 40-69 years at recruitment from the general UK population. All analyses were carried out under the application number 12505 and were conducted on both the Delta2 Server hosted by the Institute for Molecular Bioscience at the University of Queensland and the Bunya Server hosted by the University of Queensland. Participants who had withdrawn from the study were excluded (notification date 2022/02/22).

**Genotype and CNV data**

All UKB participants were genotyped at Affymetrix Research Services Laboratory (Santa Clara, California, USA) on two similar Affymetrix arrays (95% probe overlap): 49,950 samples on the Applied Biosystems UK BiLIEVE Axiom Array by Affymetrix (807,411 probes) and 438,427 samples on the Applied Biosystems UK Biobank Axiom Array (825,927 probes). Both arrays are based on the human genome reference build GRCh37/hg19. CNVs on chromosomes 1-22 were called with PennCNV v1.0.5 using PennCNV-Affy(5) by Kendall et al (8) (UKB data field 1701). The use of a single calling algorithm is accepted practice in UKB studies given the computational burden associated with its size, but we performed additional sample-level quality control. We restricted analyses to CNV calls spanning at least 10 probes, longer than 20kb, and that had a confidence score of at least 10. Similar to the ANGI CNV calls, we converted the genomic start and end coordinates of all UKB CNVs from hg19 to hg38 using the UCSC LiftOver Command Line Tool(7), requiring that 95% of the bases must remap. CNVs that failed to lift over and were dropped from further analyses.

**Sample selection**

Within the UKB, 1,260 (91.1 % female) unique individuals of inferred white European ancestry who had an AN or atypical anorexia nervosa (AAN) phenotype were categorized as AN case and 385,930 (54.6% female) as controls. The code (as used by Watson *et al*(1)) to extract the AN phenotypes from the UKB is available at https://github.com/walkeralicia/Anorexia_CNV_GWAS/blob/main/01_Samples/S1_AN_UKB_Phenotypes.R showing the field codes (from Mental Health Questionnaire, hospital inpatient and GP records) and exclusion criteria (e.g., neoplasms, Type 1 diabetes, etc.). Sample-level quality control filters, excluding those with a genotypic call rate <0.96, a waviness factor of <−0.03 and >0.03, and >30 or > 200 CNVs per person, or a LRR Sd > 0.35, as these might be indicative of genotyping errors or extreme chromosomal abnormalities. 385,797 individuals had an available BMI measurement, of which 0.3% are AN cases and 54.6% are female. Overall, 1,251 cases (91.1% female) had a recorded measure of BMI.

**Anorexia nervosa PGS**

A LOO AN GWAS summary statistics from the Watson et al.(1) study was obtained with the UKB cohort being left out. SNPs which had a low per-SNP sample size were excluded and SNPs shared between the LOO-summary stats file and the left-out cohort were retained. Polygenic score SNP weights were derived by SBayesRC. We calculated the polygenic score (PGS) of an individual in each left-out target cohort as the weighted sum of SNP allele counts, with the per allele weights for the SNPs derived from the corresponding LOO SBayesRC. AN PGS were standardized so that the cohort controls had PGS with mean zero and standard deviation of 1.

**Statistical analyses**

All CNV burden analyses conducted within ANGI (details in the main paper) was replicated within the UKB, using covariates array, sex, and age. To provide evidence that the 67 established syndromic and 178 pleiotropic disease-risk annotated rCNVs called in ANGI and UKB are approximately comparable we tested for differences in control frequency using Fisher’s Exact Test. Due to data privacy restrictions, we were unable to analyze the UKB and ANGI data in the same computing environment, hence, we used Stouffer’s method, which employs signed Z-statistics, to meta-analyze the two-sided P-values from each study. Regarding the CNV-GWAS, we tested nominally associated CNVRs from the ANGI analysis in the UKB and report results including two-sided P-values for the most significant breakpoint probe within each CNVR. The GitHub containing the pipeline to conduct a CNV-GWAS within the UKB can be found at <https://github.com/walkeralicia/Anorexia_CNV_GWAS>.

**Supplementary results: ANGI**


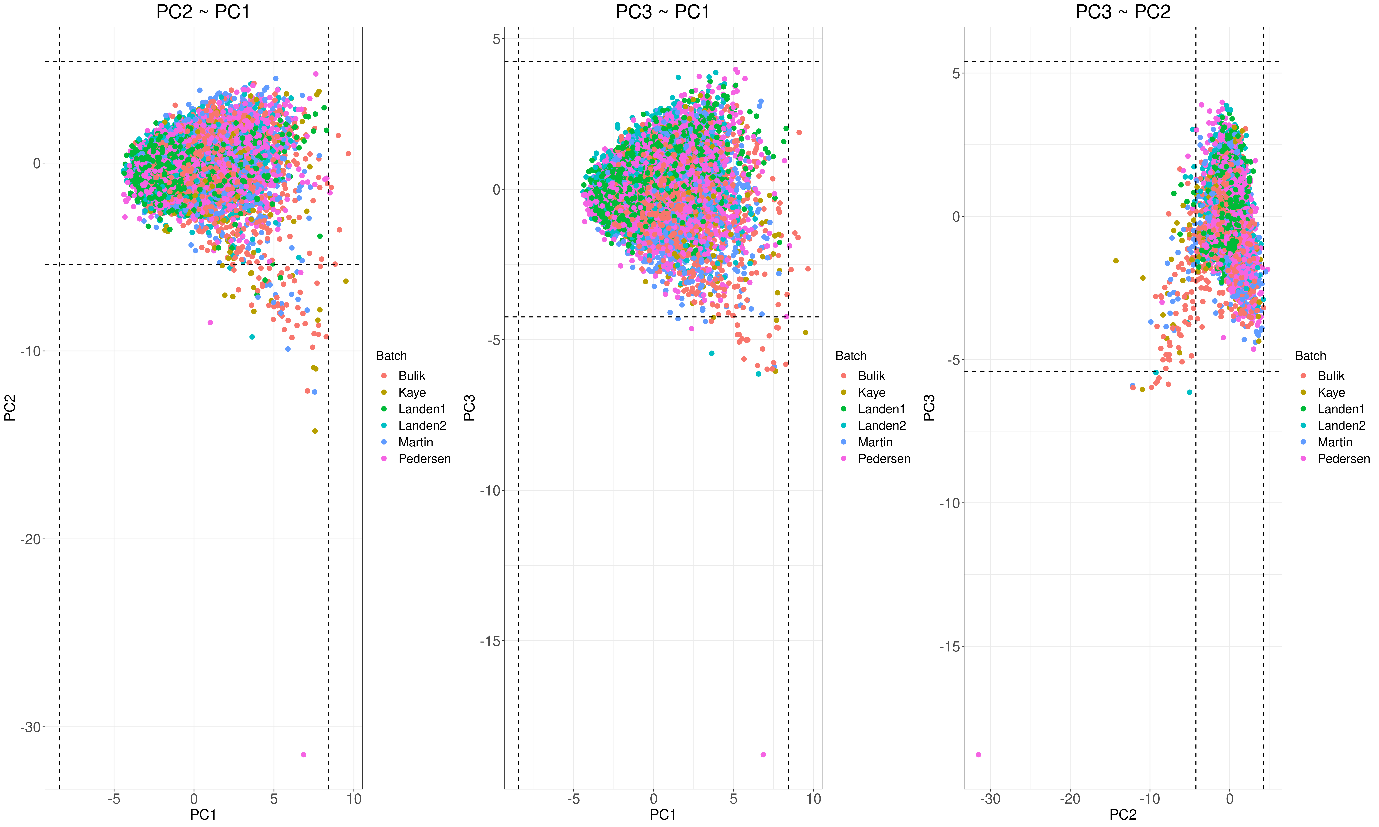


**Figure S1**. Principal Component Analysis (PCA) applied on the sample-level CNV-calling summary statistics colored by genotyping batch.


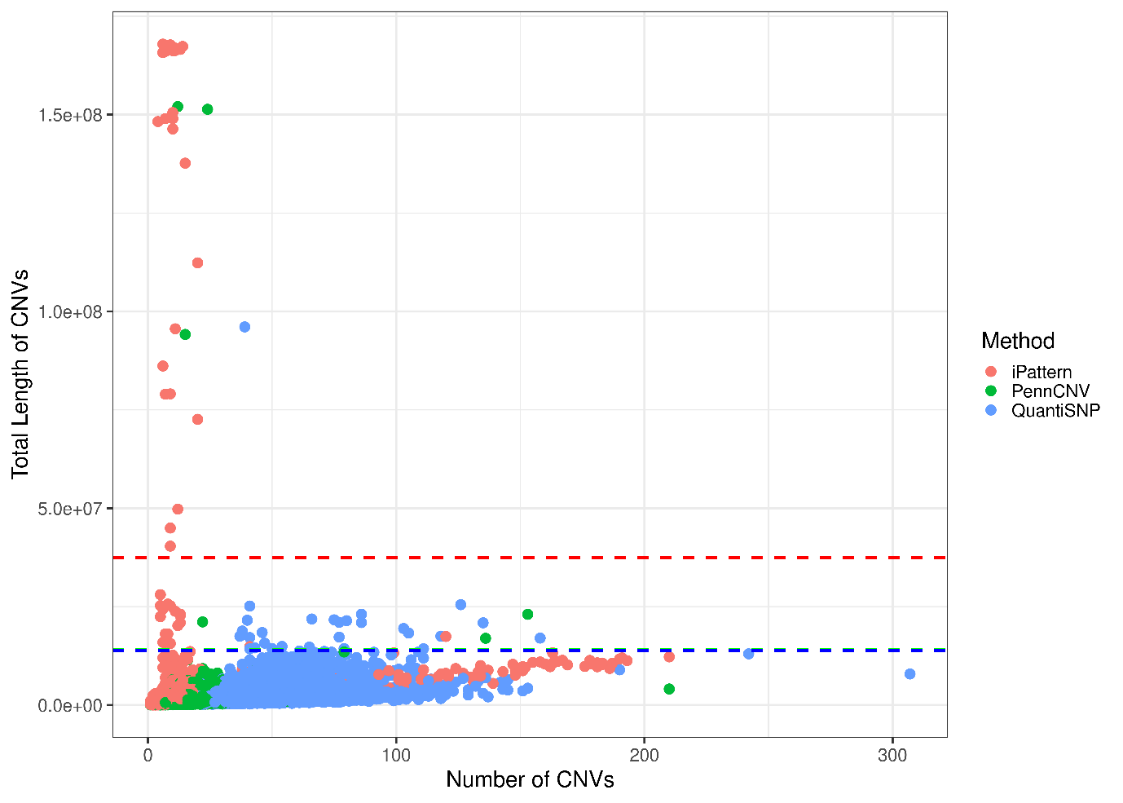


**Figure S2**: Samples with an excessive total length, defined as total CNV length greater than 6 SD from the median within each CNV calling algorithm.


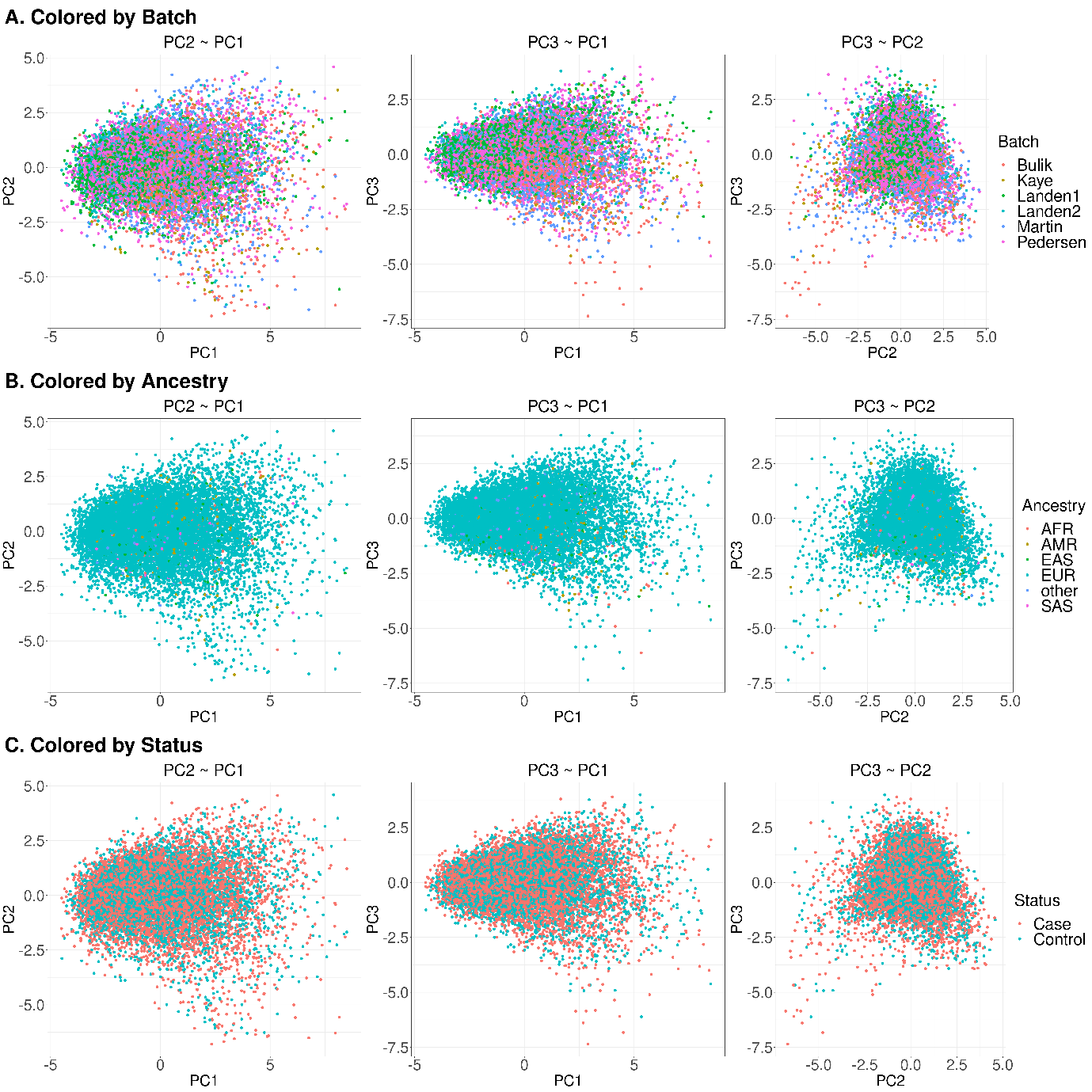


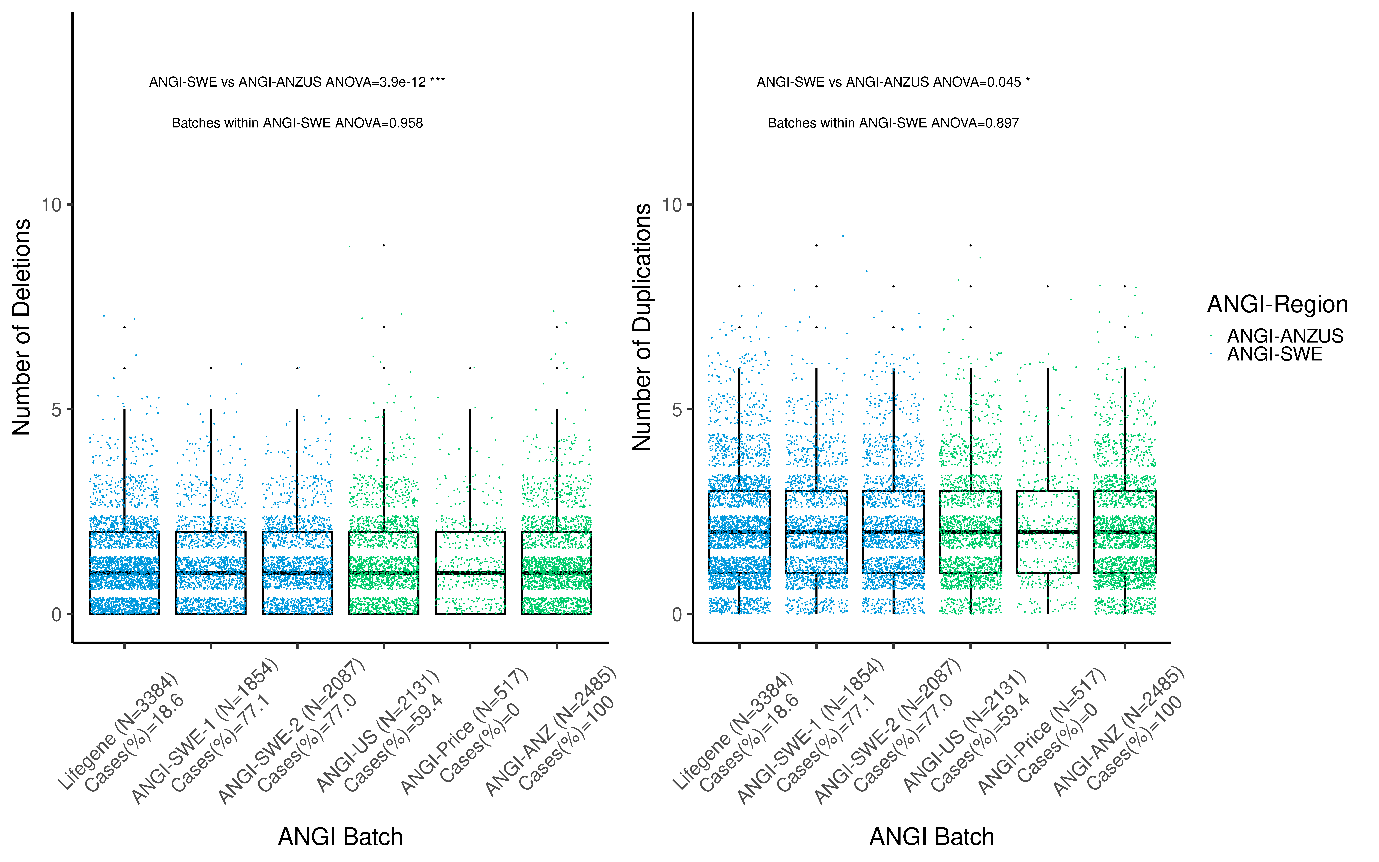
**Figure S3:** Reran PCA on the sample-level summary statistics for the 12,675 individuals that passed QC, and present a PC2 vs PC1 plot with each individual colored by batch, AN status, or PCA-inferred ancestry.

**Figure S4**: CNV rates across batches, split by CNV-type with one-way non-parametric ANOVA tests to identify differences in CNV rates between regional cohorts (ANGI-SWE/ANGI-ANZUS) and within ANGI-SWE batches.


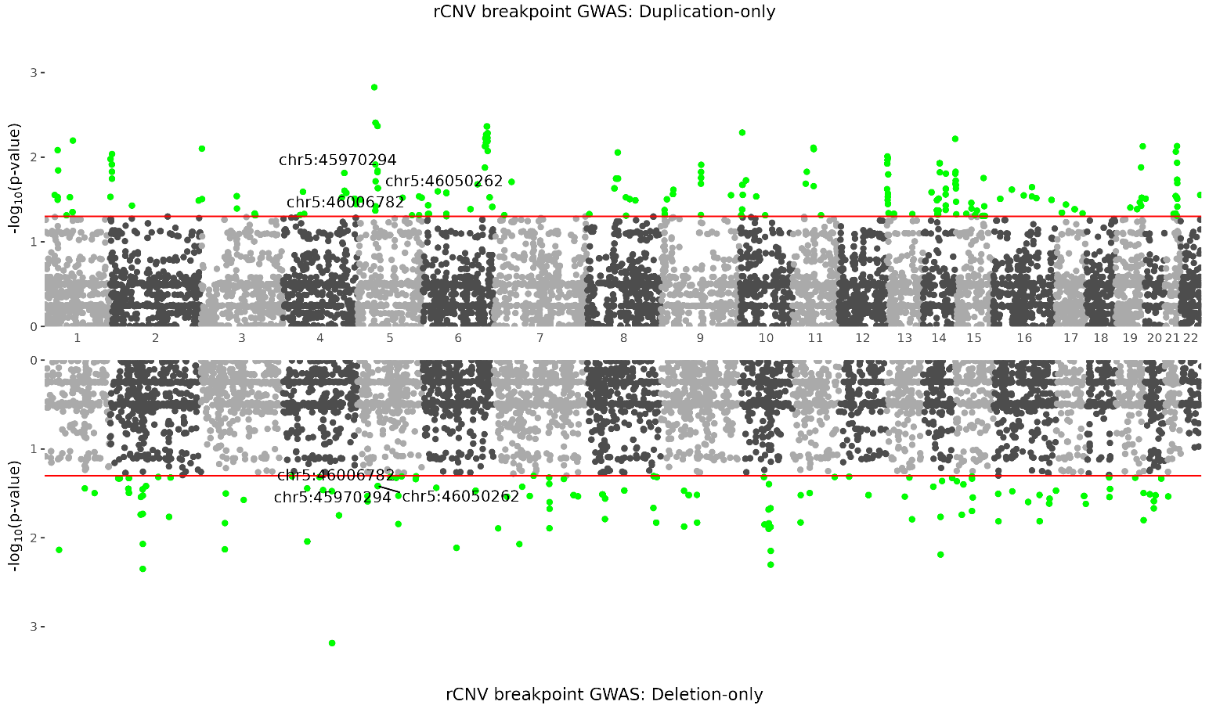


**Figure S5:** ANGI SAIGE results for rCNV breakpoint associations across autosomes in a duplication-only and a deletion-only model. Three loci located on cytoband 5p12 (hg38) with a “mirror” effect are annotated.


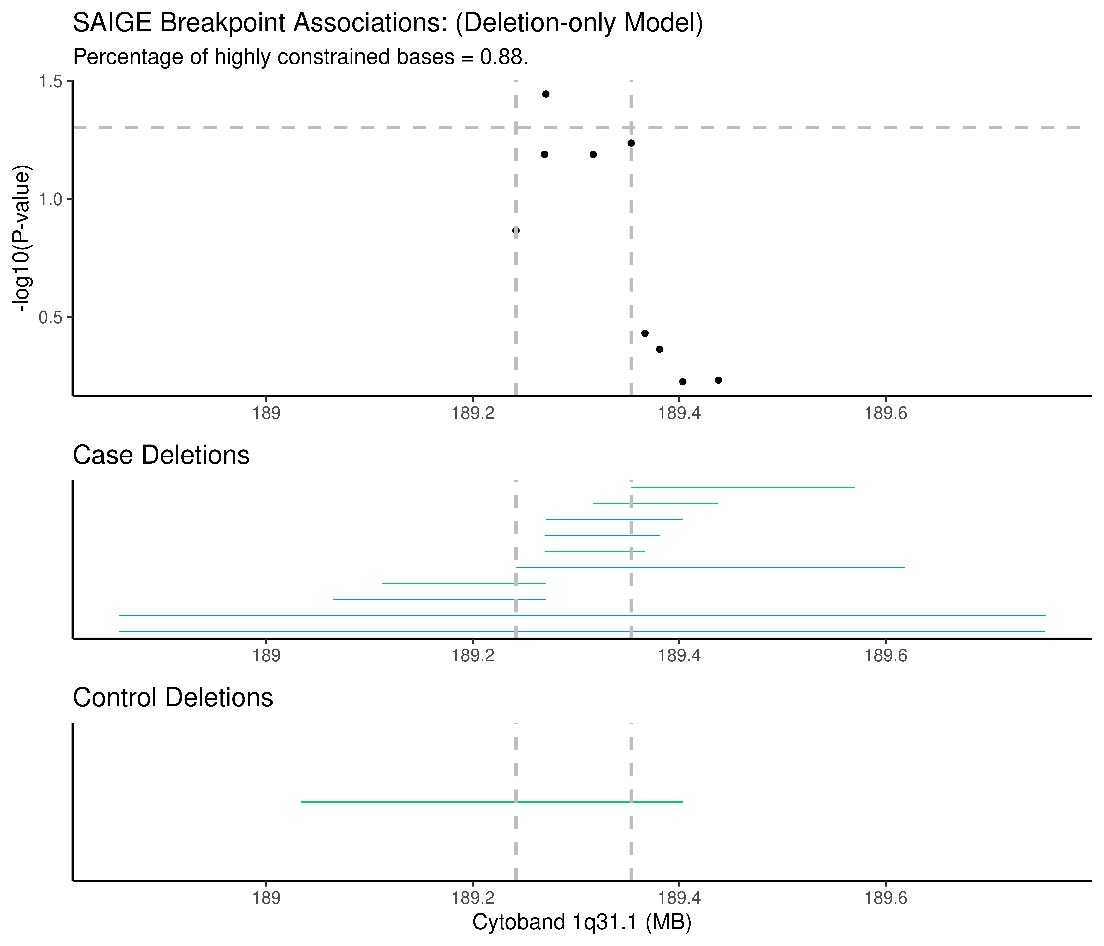
**Figure S6.01-10.20:** ANGI rCNV breakpoint association results for nominally significant AN-risk CNV regions identified via SAIGE CNV-type specific rCNV breakpoint analyses. The two vertical grey dashed lines within all panels illustrate the critical region of the AN-risk CNVR. The top panel illustrates SAIGE rCNVb associations from the corresponding CNV-type specific model within the defined critical CNVR boundary and a buffer region corresponding to the length of the CNVR. Gene tracks panel shows the intersecting protein-coding gene tracks extracted from the UCSC genome browser (v43). The bottom two panels illustrate the tracks for CNVs found within AN cases and controls intersecting the CNVR by at least 1 base-pair, with green CNVs indicating individuals from ANGI-ANZUS, and blue CNVs indicating individuals from ANGI-SWE. An individual may have greater than 1 CNV within the region. The number of CNVs per cohort contributes to the differences in the test statistic and -log10 p-value across the x-axis.

**Figure S6.01:** chr1:189241900-189353477 (1q31.1) deletion.


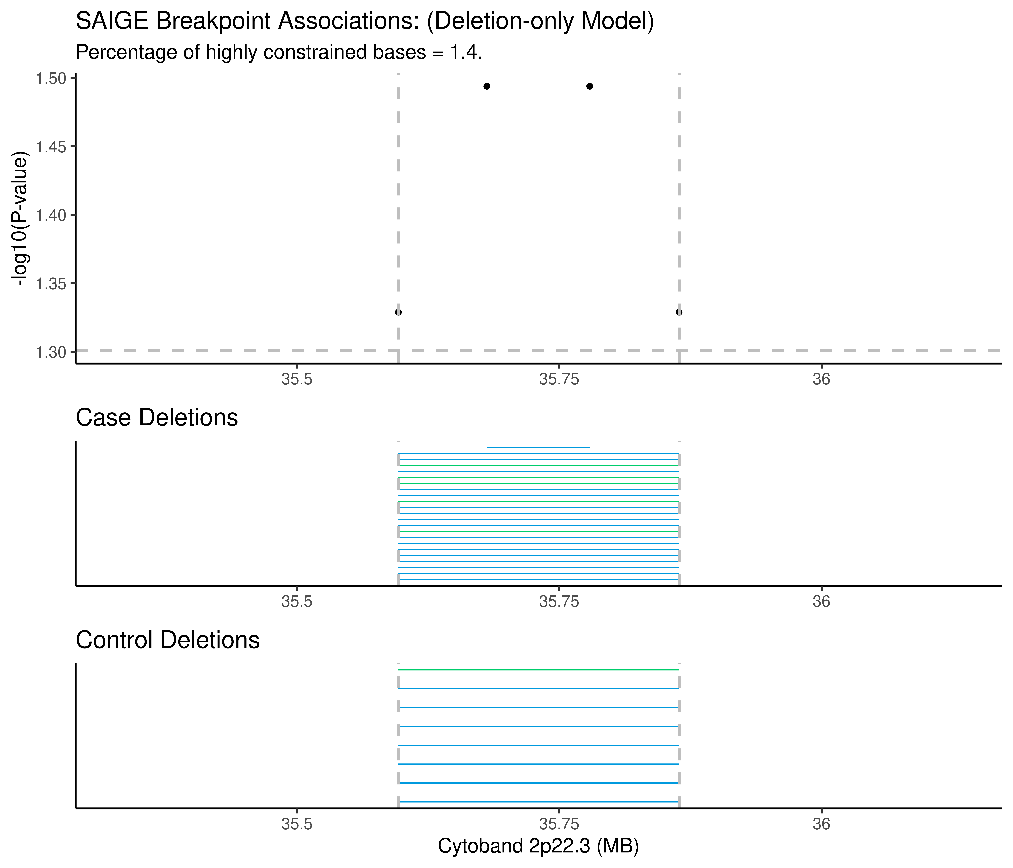


**Figure S6.02:** chr2:35596644-35864166 (2p22.3) deletion.


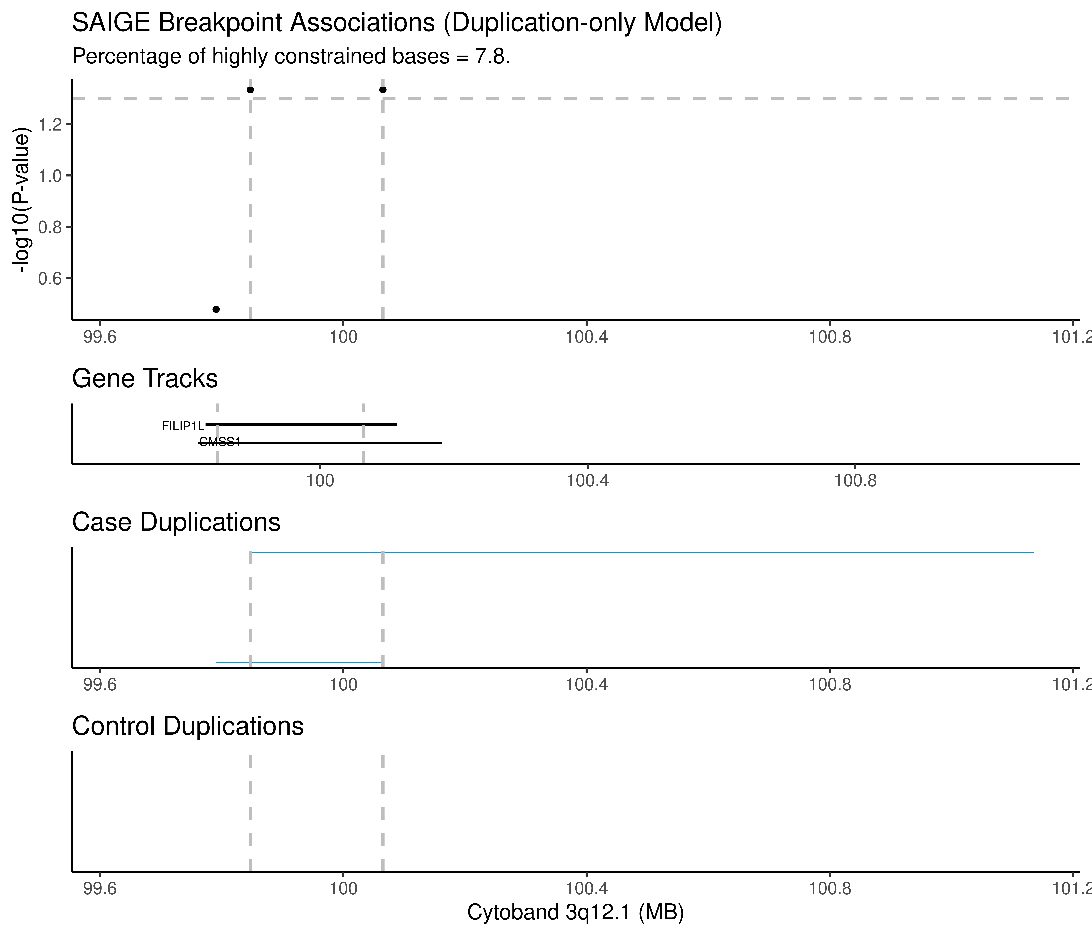


**Figure S6.03:** chr3:99847014-100065076 (3q12.1) duplication intersecting the *FILIP1* and *CMSS1* genes.


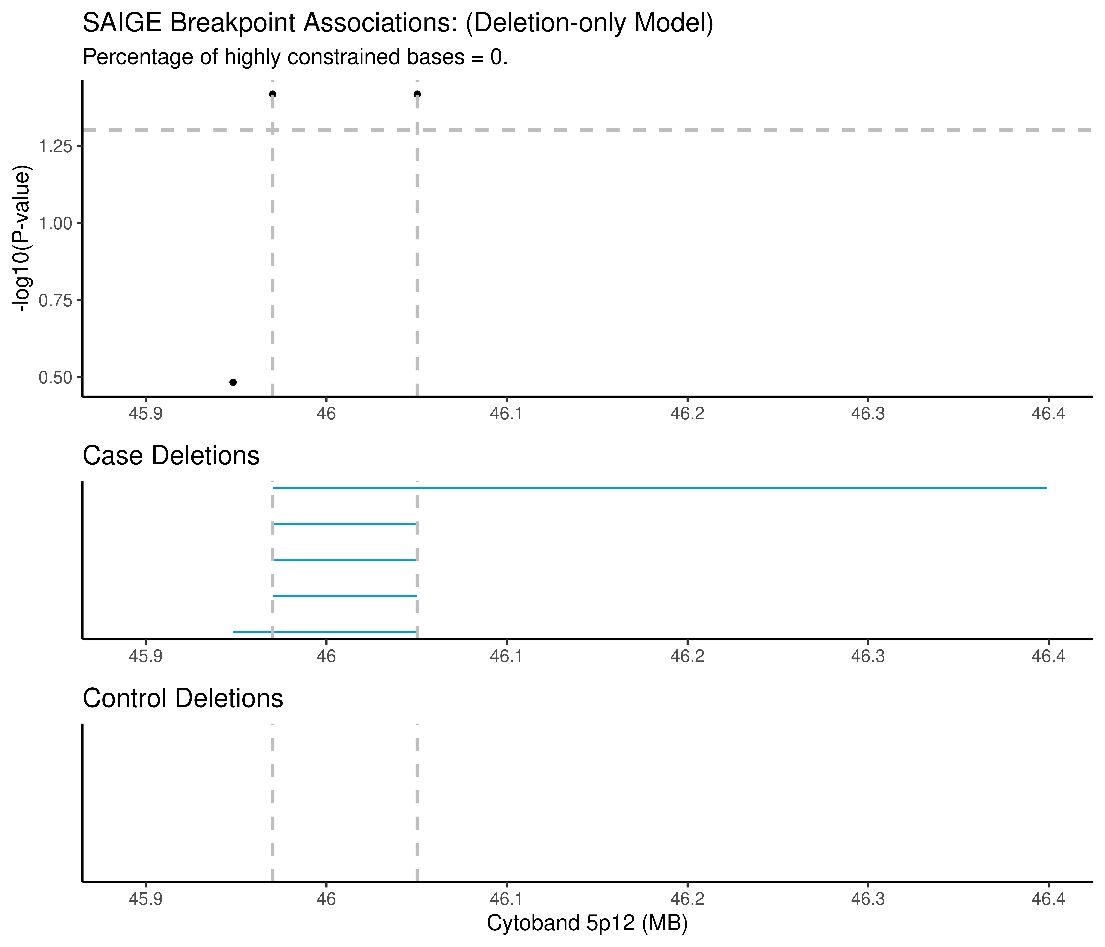


**Figure S6.04:** chr5:45970294-46050262 (5p12) deletion.


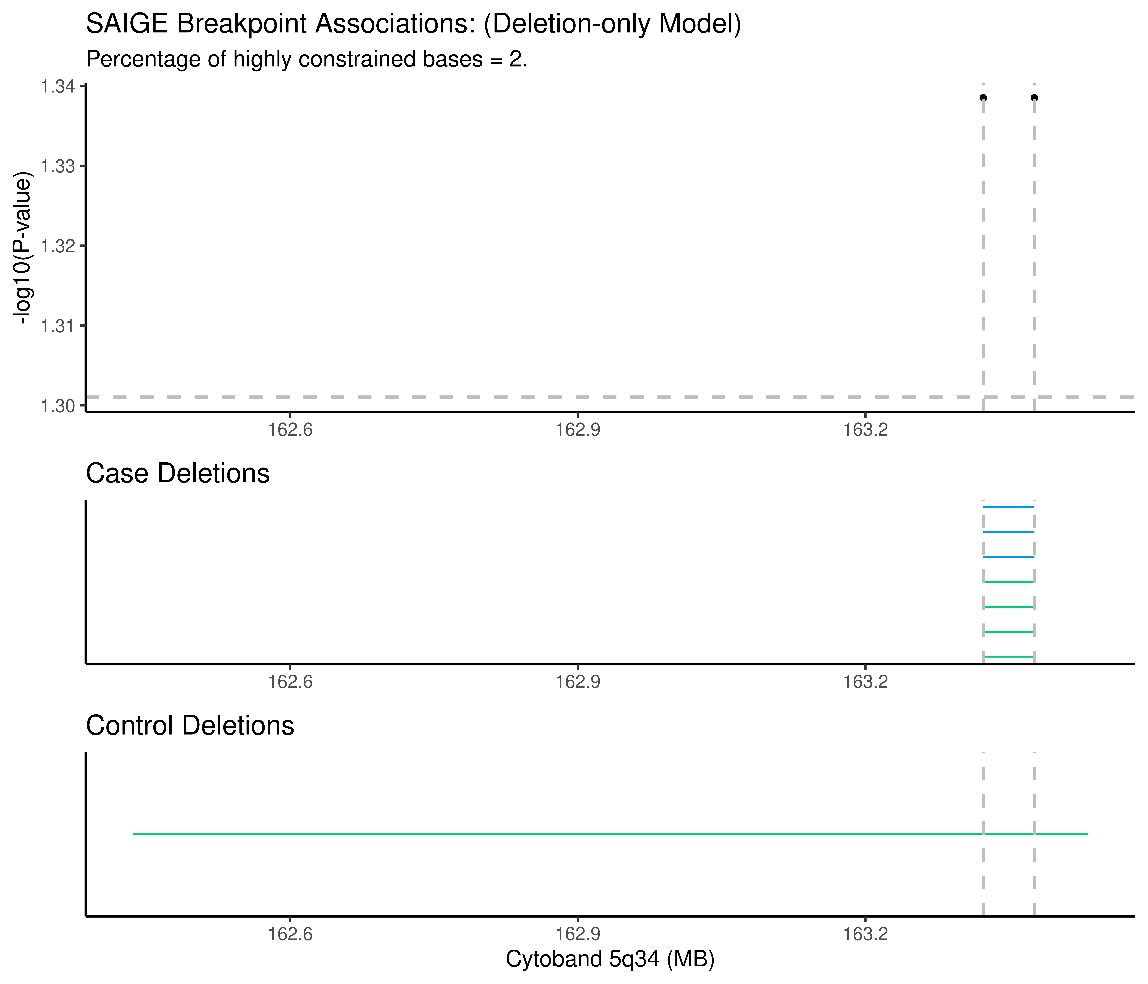


**Figure S6.05:** chr5:163322967-163376252 (5q34) deletion.


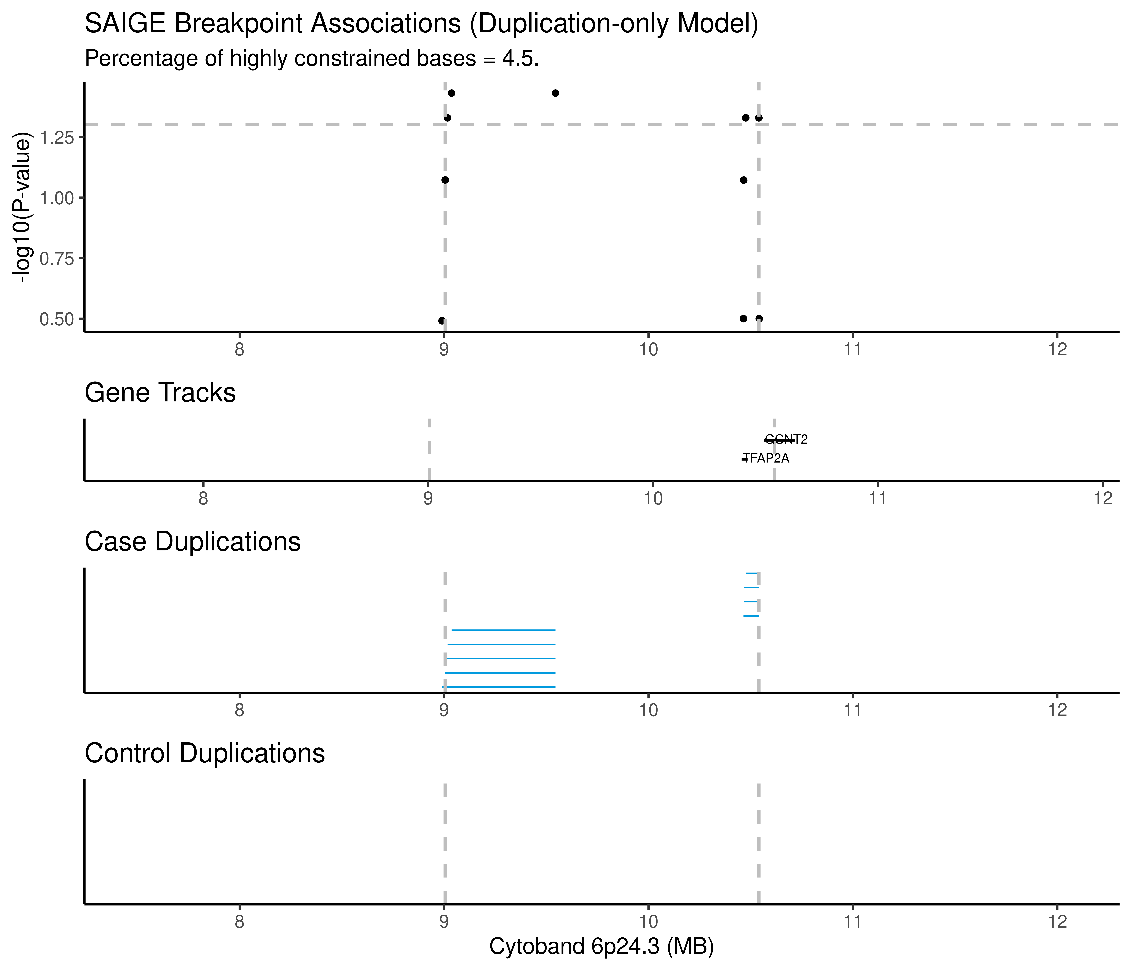


**Figure S6.06:** chr6:9005377-10539744 (6p24.3) duplication intersecting the *GCNT2* and *TFAP2A* genes.


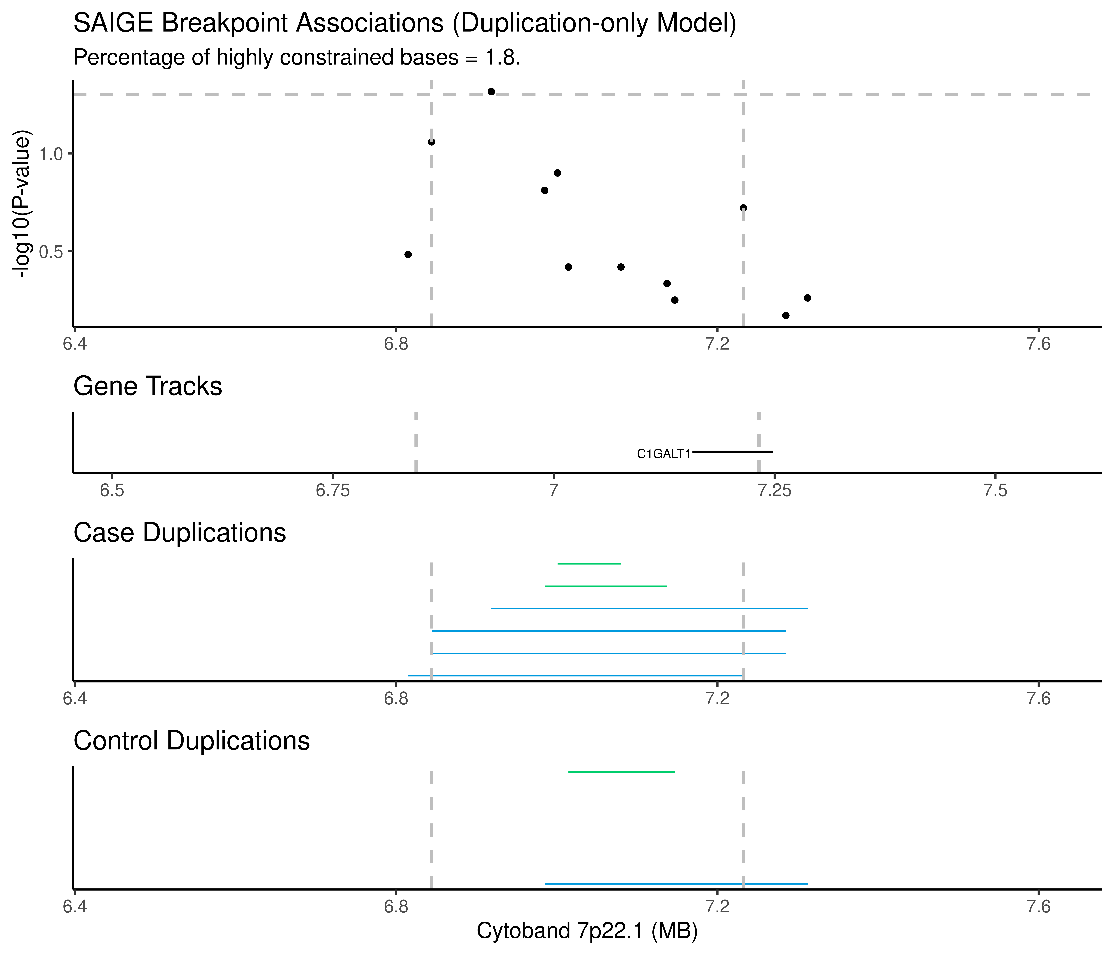


**Figure S6.07:** chr7:6844111-7232248 (7p22.1) duplication intersecting the *C1GALT1* gene.


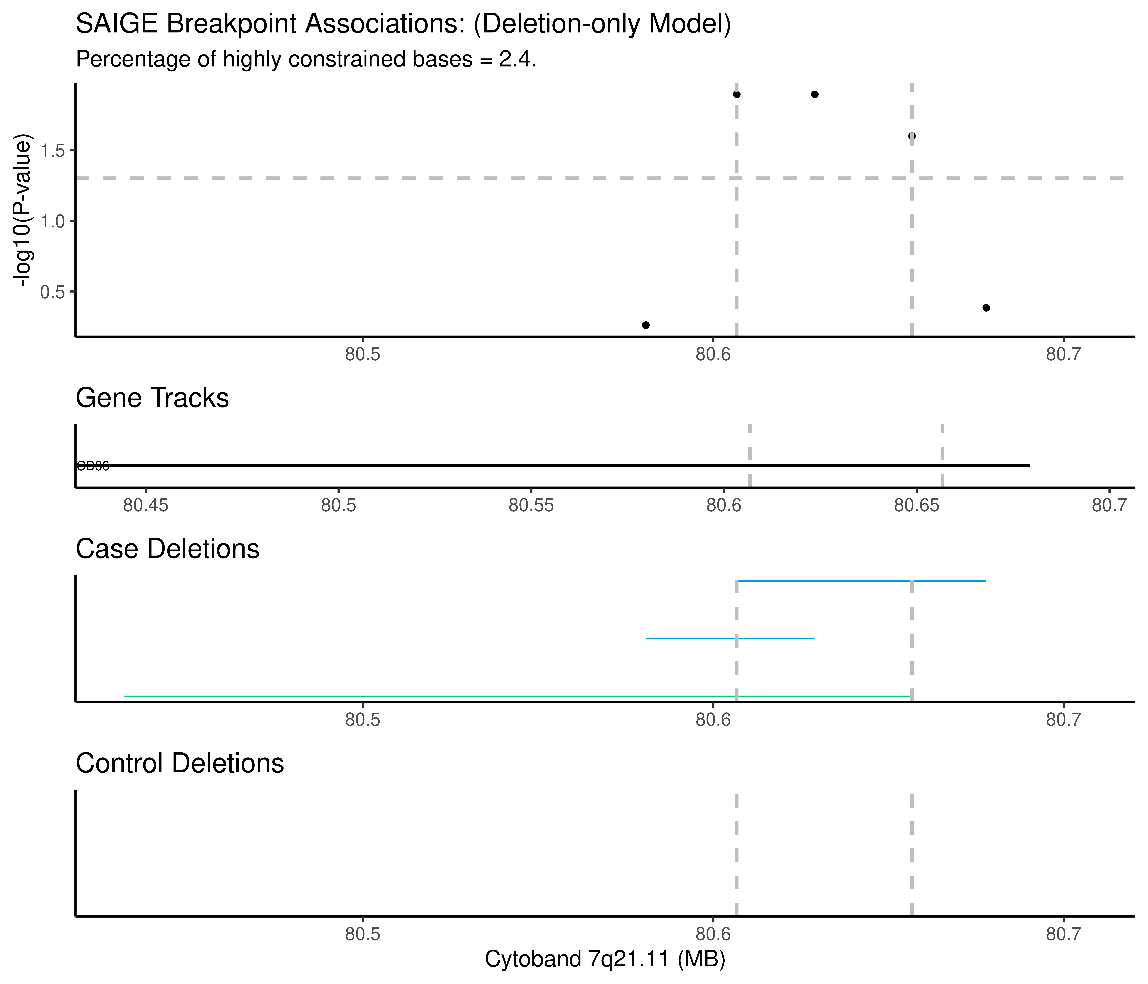


**Figure S6.08:** chr7:80606698-80656701 (7q21.11) deletion intersecting the *CD36* gene.


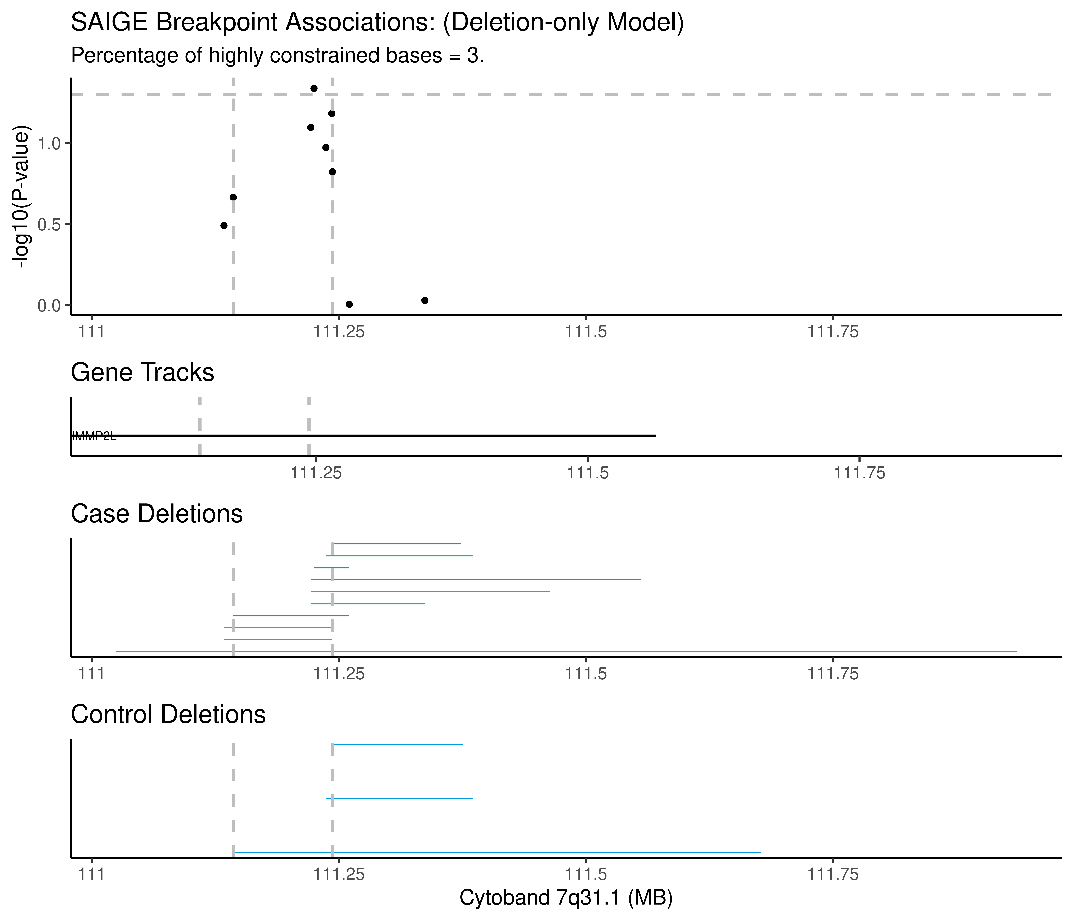


**Figure S6.09:** chr7:111143168-111243508 (7q31.1) deletion intersecting the *IMMP2L* gene.


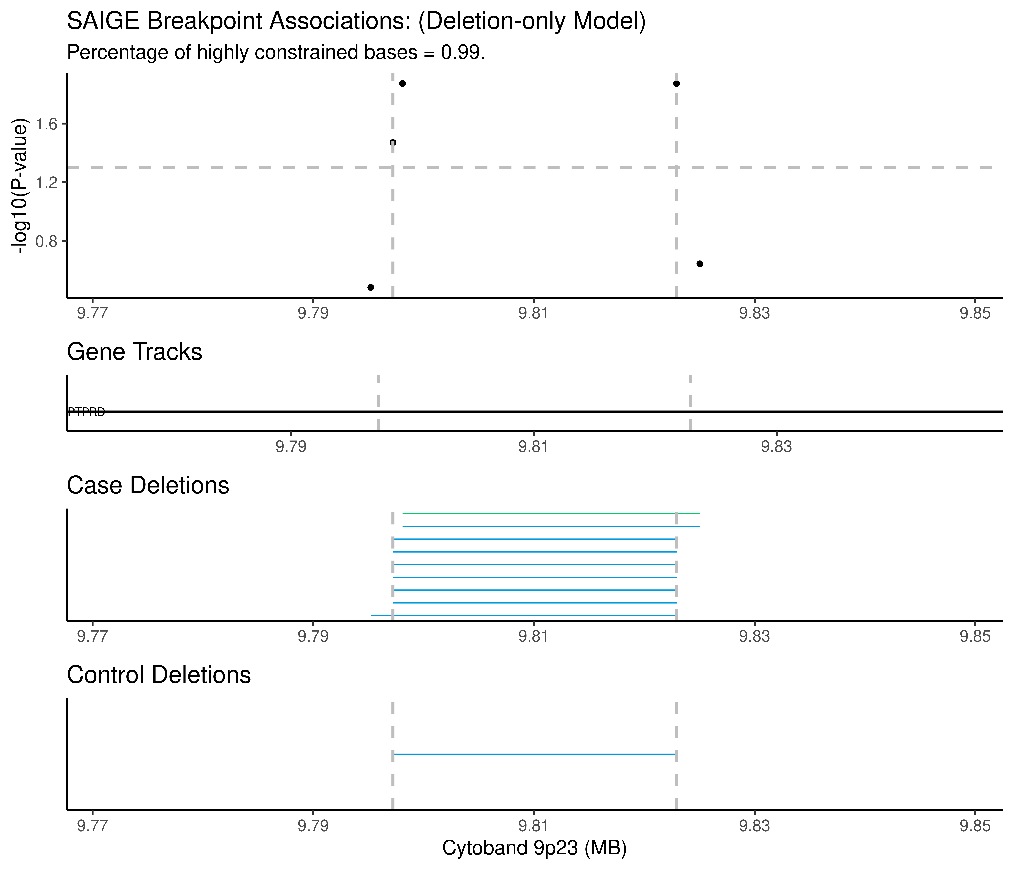


**Figure S6.10:** chr9:9797221-9822930 (9p23) deletion intersecting the *PTPRD* gene.


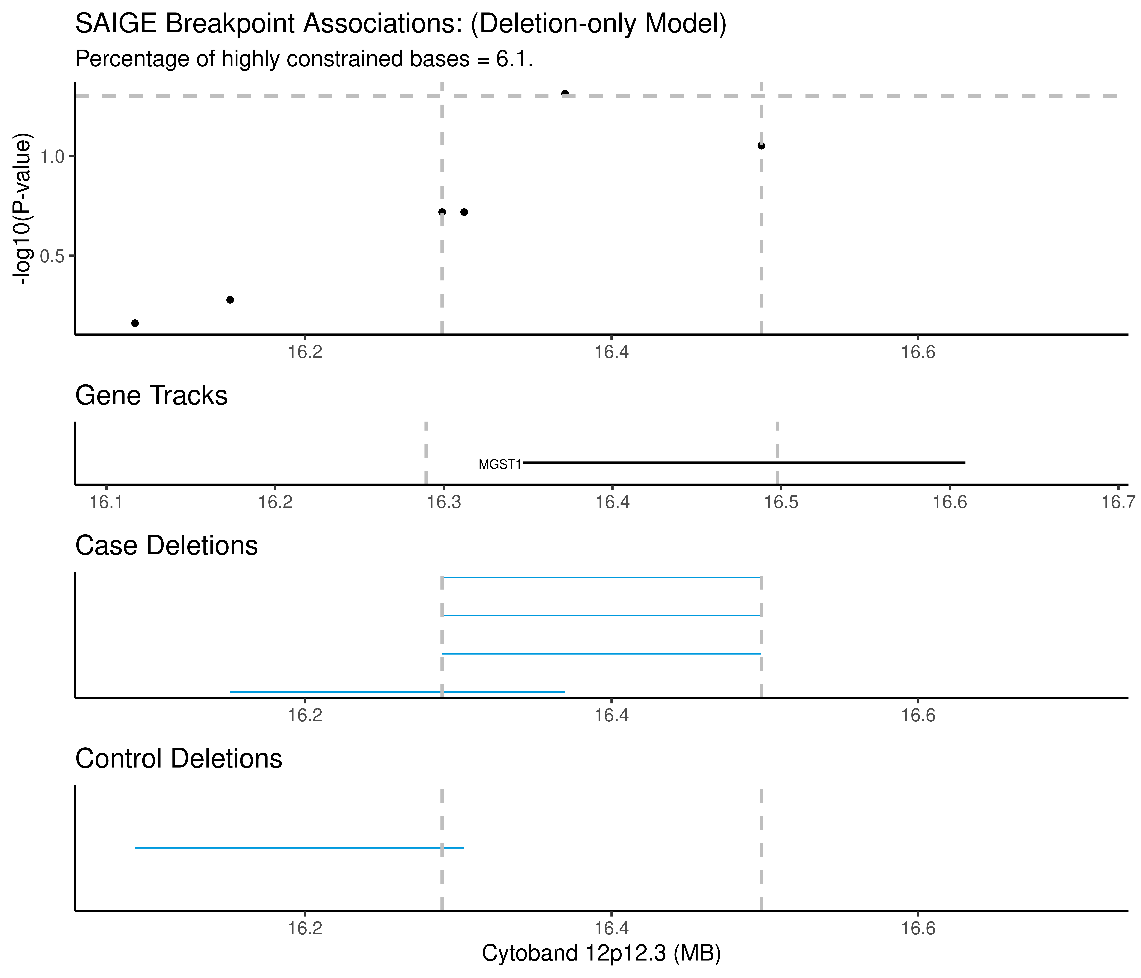


**Figure S6.11:** chr12:16289542-16497734 (12p12.3) deletion intersecting the *MGST1* gene.


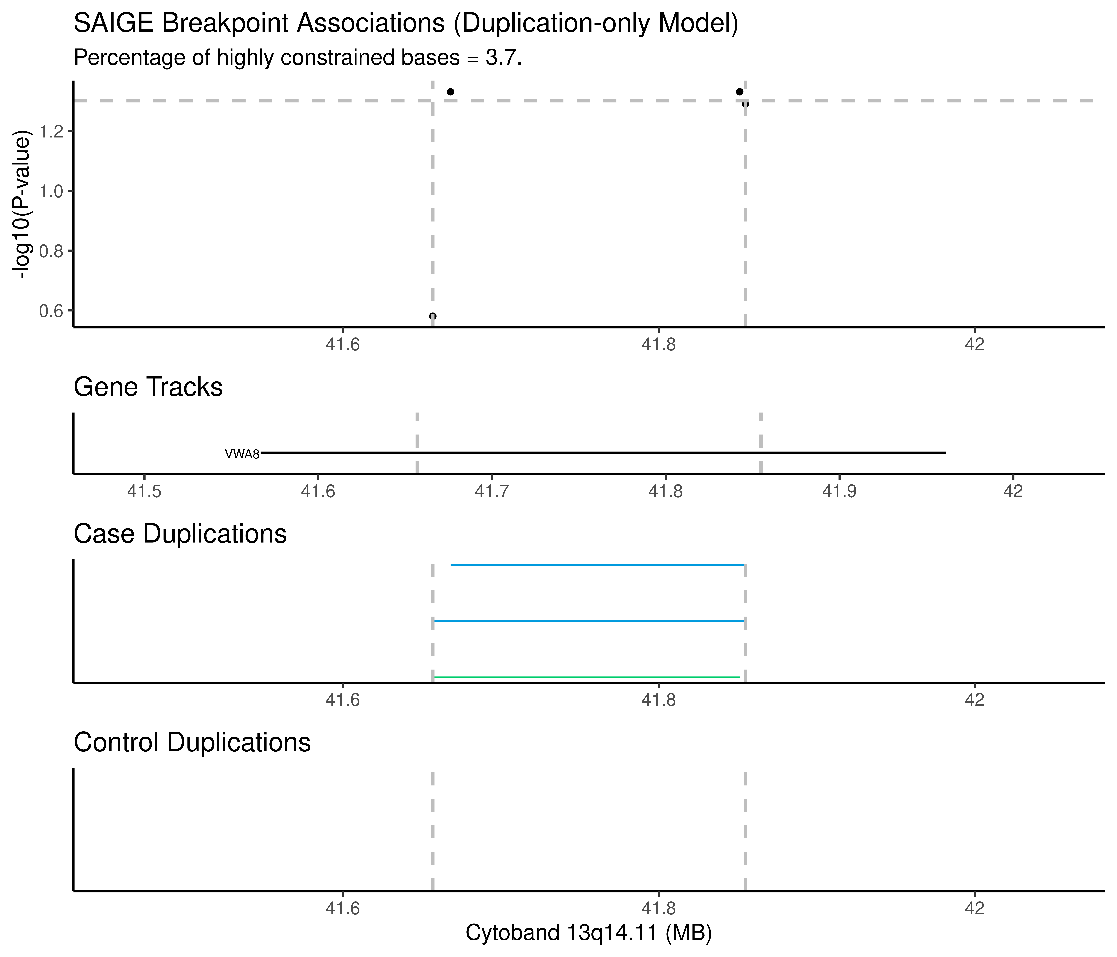


**Figure S6.12:** chr13:41656953-41854763 (13q14.11) duplication intersecting the *VWA8* gene.


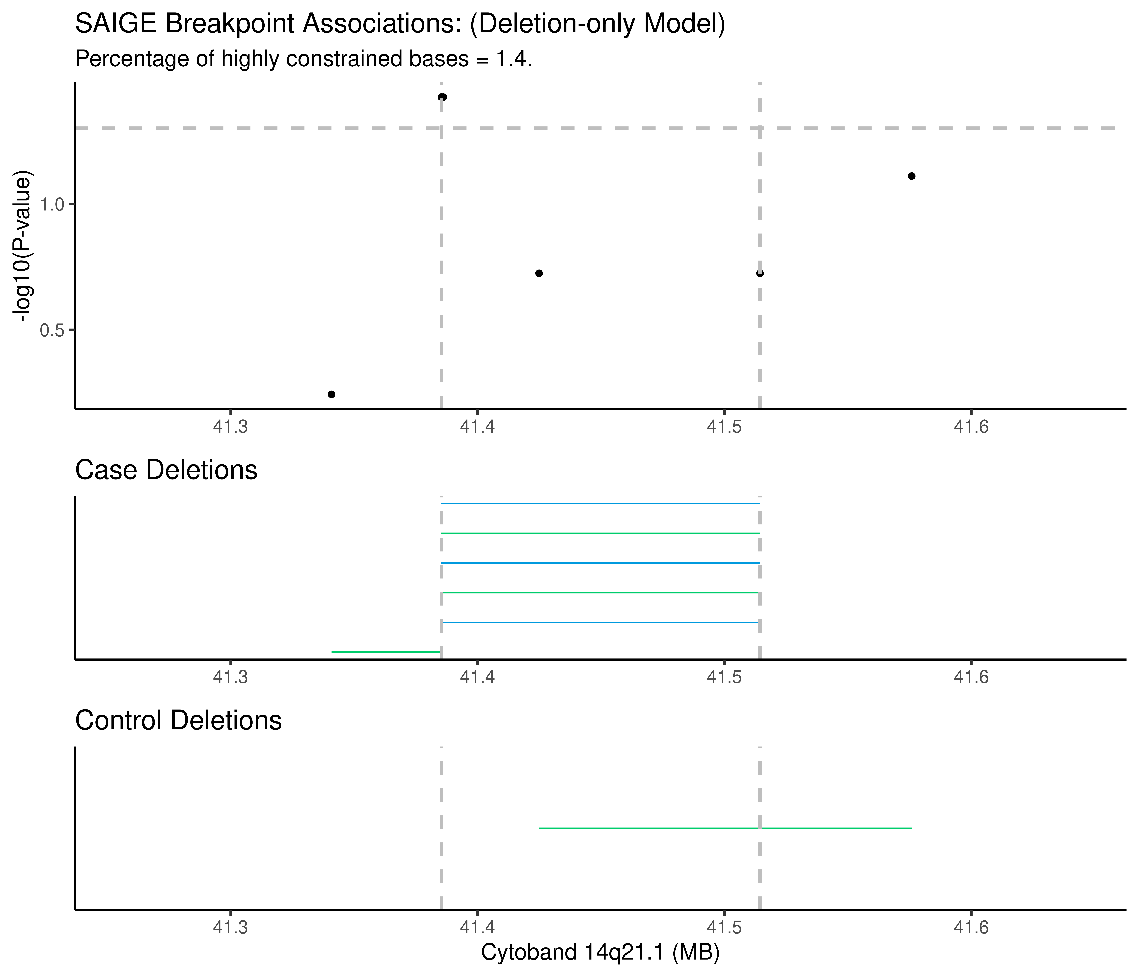


**Figure S6.13:** chr14:41385360-41514385 (14q21.1) deletion.


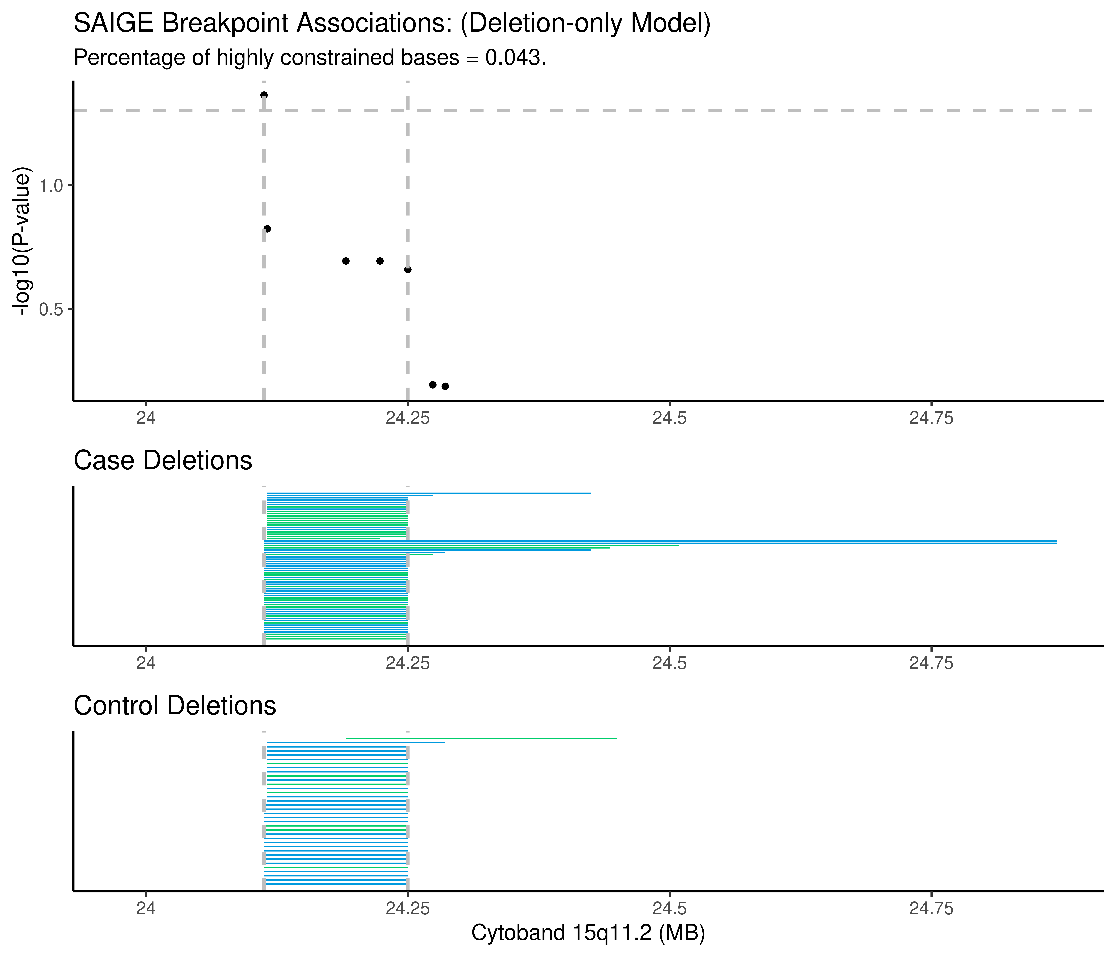


**Figure S6.14:** chr15:24112578-24249920 (15q11.2) deletion.


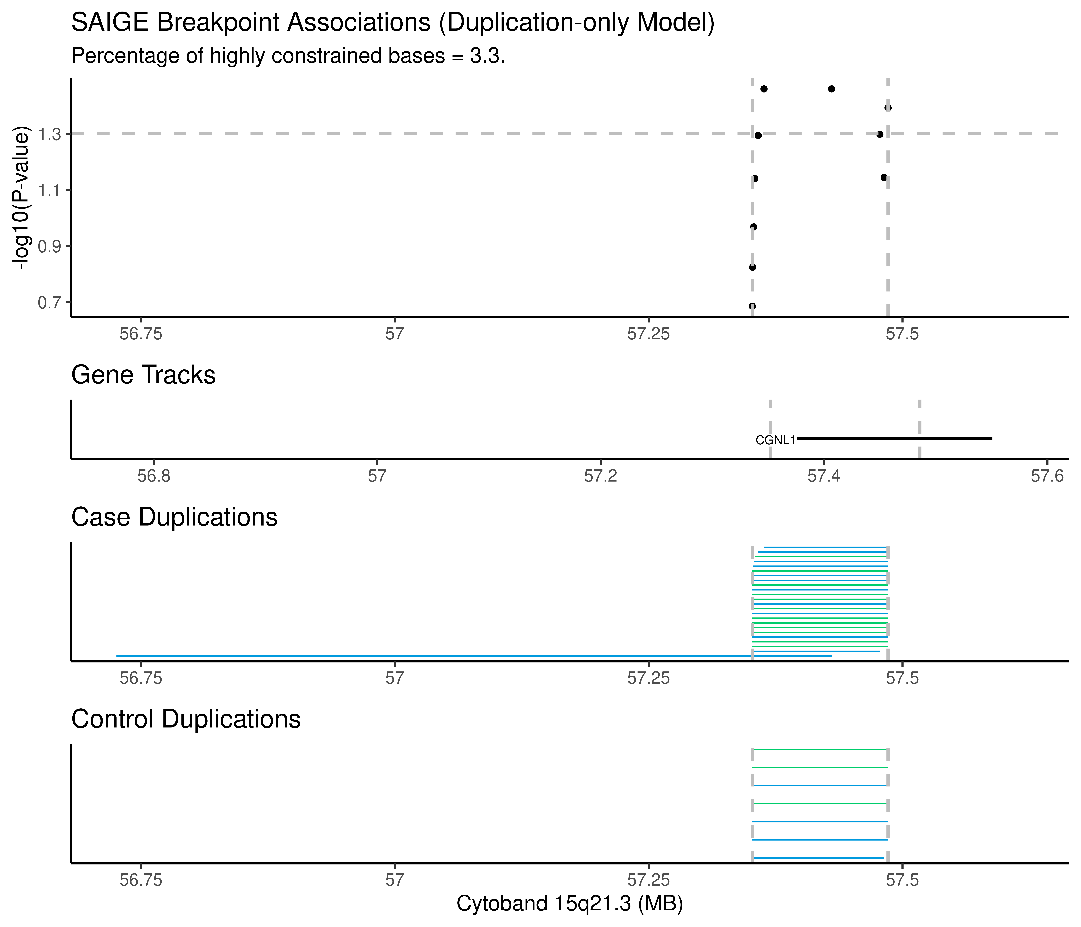


**Figure S6.15:** chr15:57352069-57485748 (15q21.3) duplication intersecting the *CGNL1* gene.


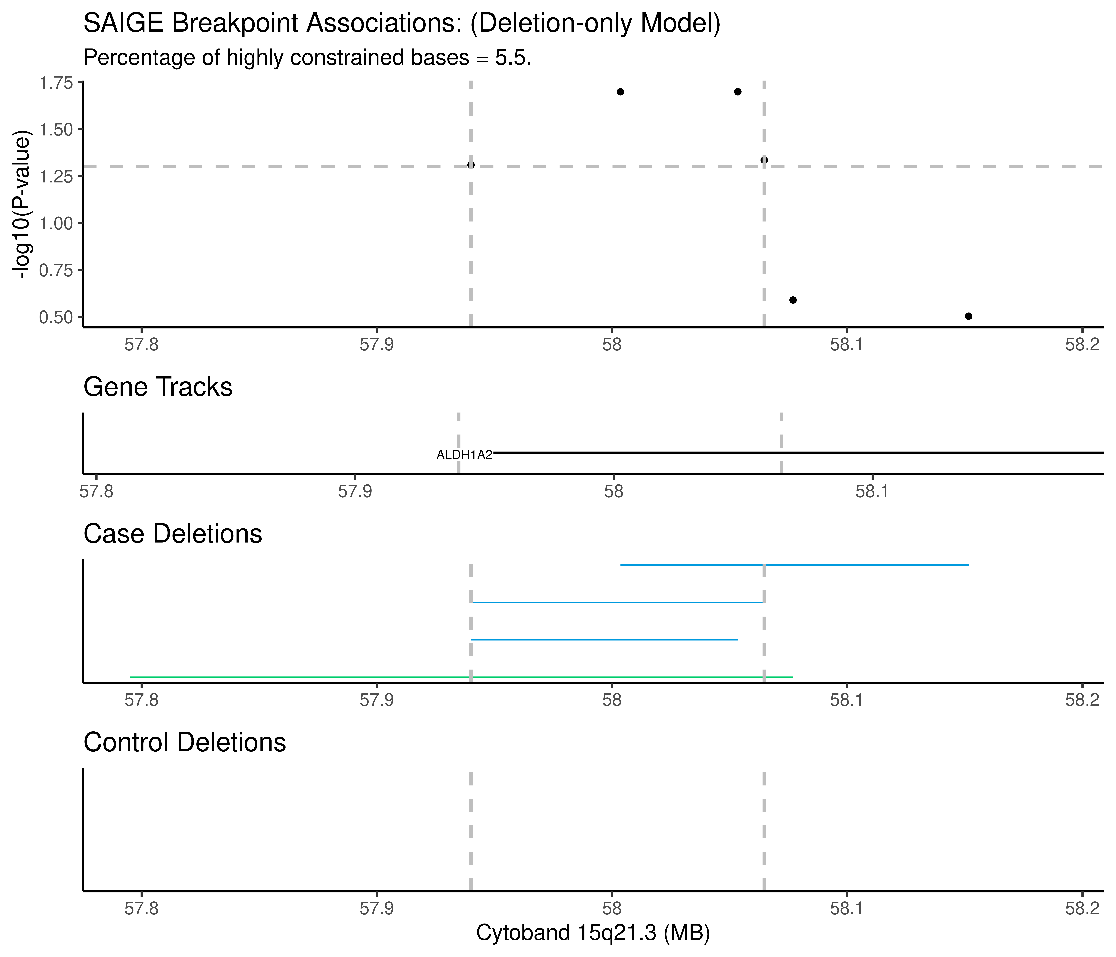


**Figure S6.16:** chr15:57940038-58064676 (15q21.3) deletion intersecting the *ALDH1A2* gene.


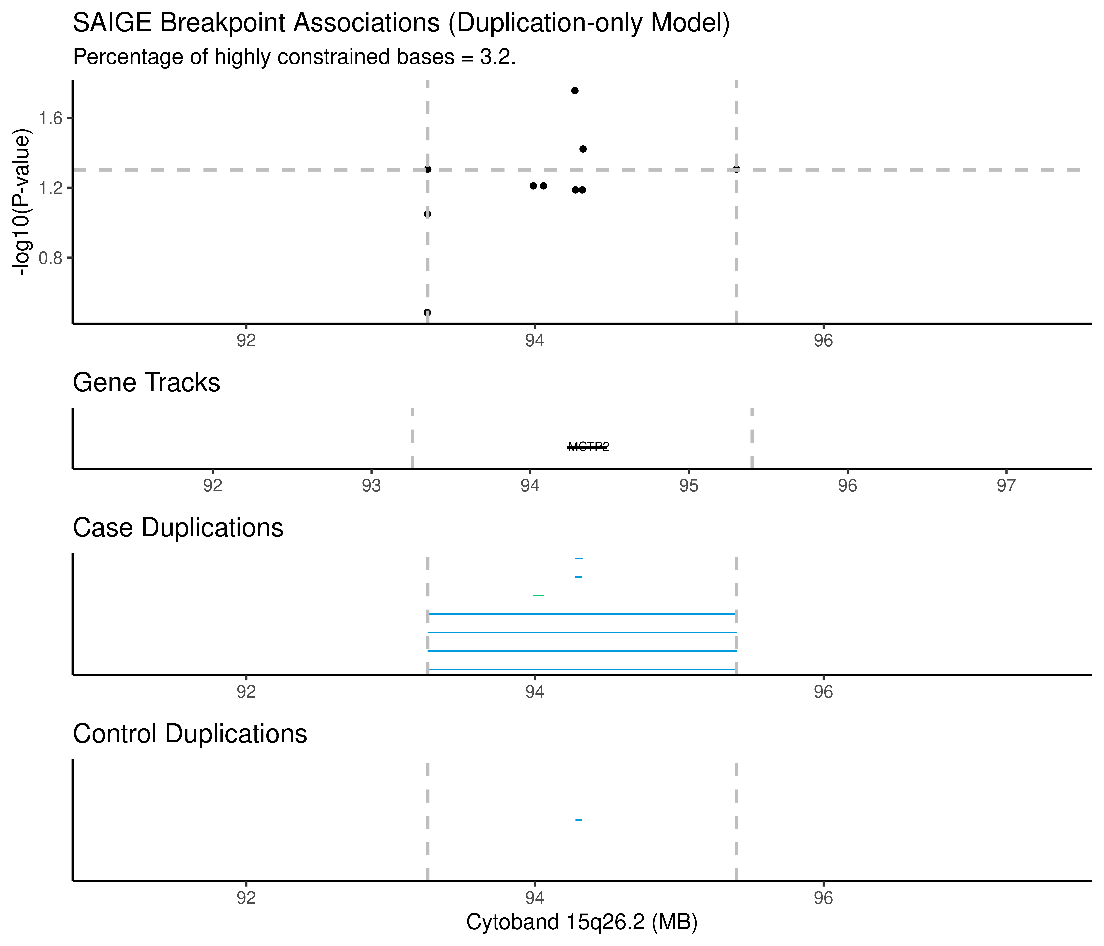


**Figure S6.17:** chr15:3257196-95397582 (15q26.2) duplication intersecting the *MCTP2* gene.


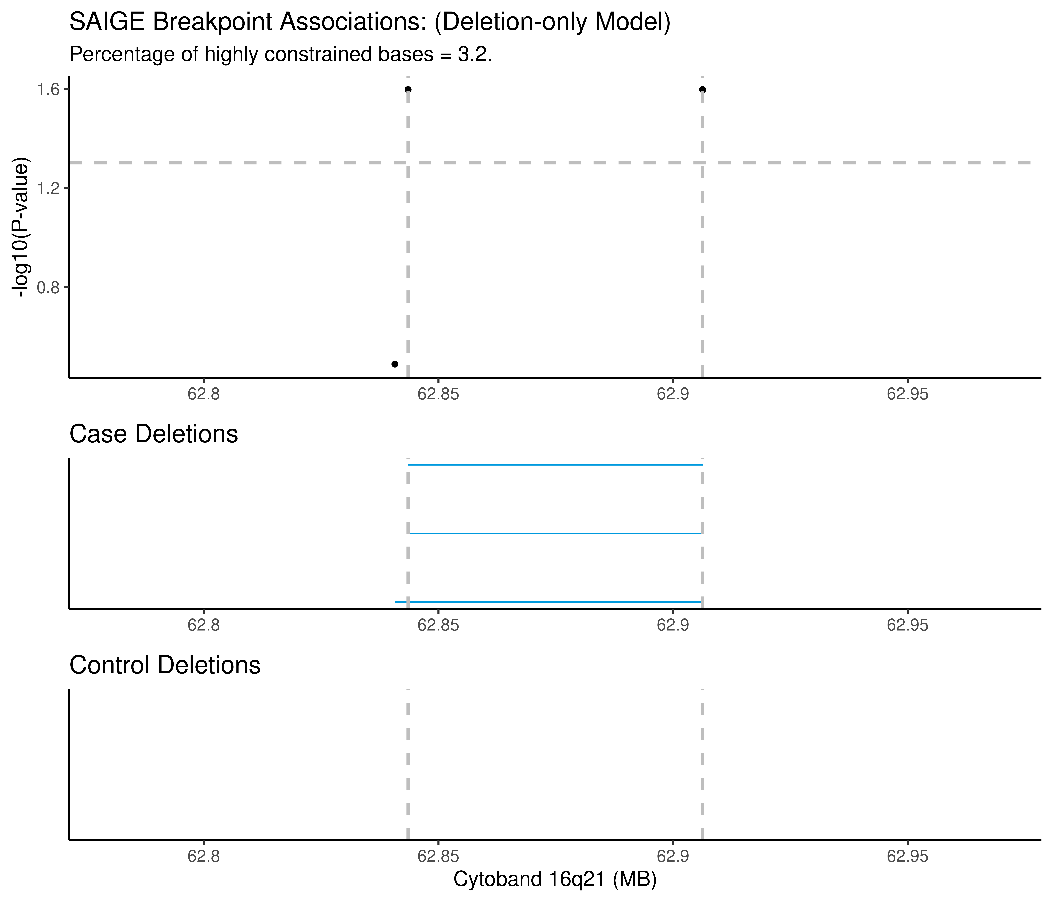


**Figure S6.18:** chr16:62843514-62906295 (16q21) deletion.


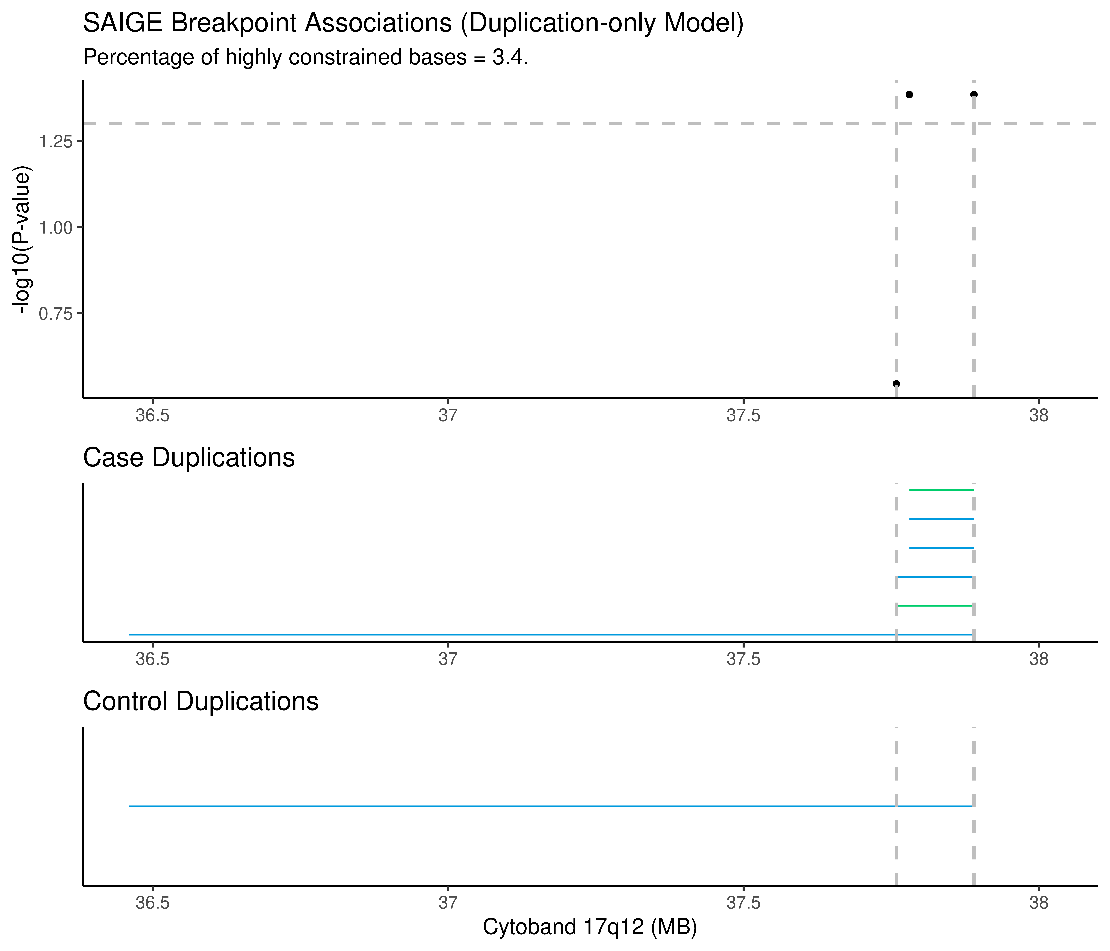


**Figure S6.19:** chr17:7758297-37889808 (17q12) duplication.


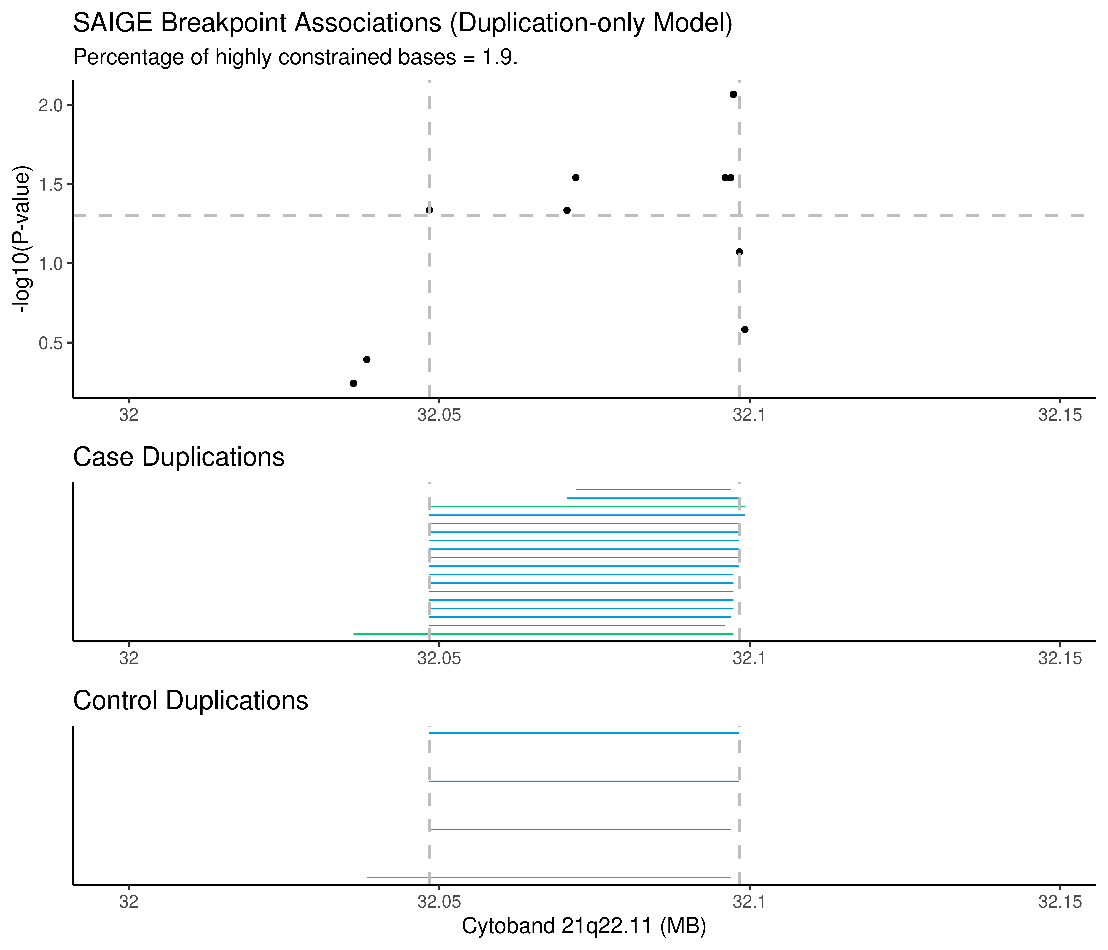


**Figure S6.20:** chr21:32048392-32098317 (21q22.11) duplication.

**Supplementary results: The UKB and meta-analyses with ANGI**

**Total rare CNV burden**

For the UKB, we used a set of 1,755,168 CNVs over 387,190 samples consisting of 1,260 AN (91% female) cases and 385,930 (55% female) controls. For rare CNVs (N=674,425) the median size was 80.0kb and an average of 2.2 CNVs (1.3 deletions and 0.9 duplications) per person.

Similar to ANGI, total rCNV burden was not greater among AN cases than controls within the UKB when measured as total distance covered with every 100kb or when measured as total CNV count (***Table S3***). Moreover, the burden of rCNVs affecting genes with dosage sensitivity (9) showed no enrichment of deletions affecting haploinsufficient genes and no enrichment of duplications affecting triplosensitive genes (***Table S3)***. Further analysis examining genome-wide rCNV burden across length range (20-100kb to >500kb) and frequency range (singletons to 1% frequency), as well as split by CNV type, did not reveal any genome-wide significant enrichment associated with AN (***Figure S7***). A marginal excess of 20-100kb rCNVs was seen in UKB AN cases relative to controls (OR: 1.23, CI: 1.04-1.45, one-sided P: 0.007), though not reaching genome-wide significance. Partitioning by CNV type, this suggestive excess was mostly due to deletions (OR: 1.1, CI: 0.97-1.26) than duplications (OR: 1.02, CI: 0.91-1.4) (***Figure S7***).


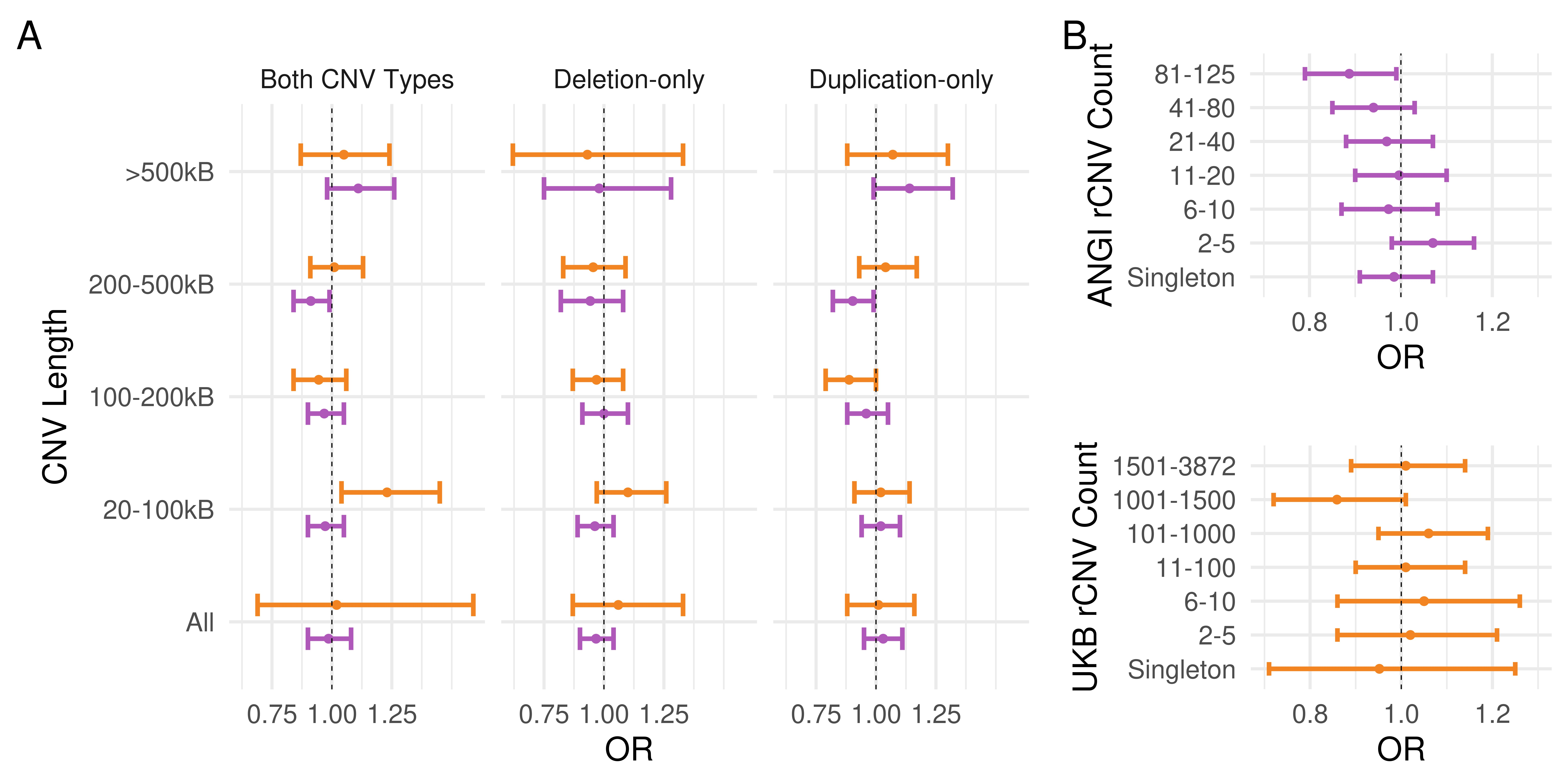


**Figure S7: Net genome-wide rare CNV (rCNV) burden in ANGI and the UKB.** We tested whether genome-wide rCNV burden was greater in AN cases than in controls in ANGI (purple) and the UKB (orange) partitioned across CNV types and CNV lengths (A), as well as CNV counts, ranging from singletons up to 1% population frequency (B).

**Syndromic CNV associations with AN**

Of the 67 annotated syndromic CNVs, 9 (13%) CNVs had no observations within the UKB. A simple test of differences in control frequencies between the UKB and ANGI showed no significant differences (P > 0.05/67) for 58 out of the 67 loci (87%).

Among the 67 examined loci, the 3q29 (3:196-198MB) duplication syndrome CNV exhibited nominal significance following meta-analysis of the two datasets (***Table S4***, P = 0.014). This CNV was linked to an increased risk of AN, driven mainly within the UKB (OR: 4.96, CI: 1.86-10.54, P = 0.0032) rather than ANGI (OR: 1.4, CI: 0.4-7.7, P = 0.60). These 3q29 duplications, though rare, were more frequent in UKB AN cases (0.4%) compared to ANGI cases (0.09%). However, there was no significant difference in CNV frequencies among controls in the two datasets (P = 0.34, UKB = 0.09%, ANGI = 0.04%). Despite a syndromic region of ~1.62 Mb, average CNV lengths annotated to the region were only ~75kb in ANGI and ~212kb in the UKB. In ANGI cases, seven 3q29 duplications affecting at least one protein-coding gene were identified, contrasting with one control case with gene disruption (***Figure S7***). While the clinical significance of 3q29 duplications is unclear, they have been associated with failure to thrive and mild-moderate ID(10, 11). In addition, among 31 individuals from the 3q29 registry with 3q29 duplications, 55% have been reported to experience feeding problems, and 44% had difficulty gaining weight(12). Conversely, reciprocal deletions have been implicated in a range of neuropsychiatric disorders, including bipolar disorder (BP)(13) and schizophrenia(14).


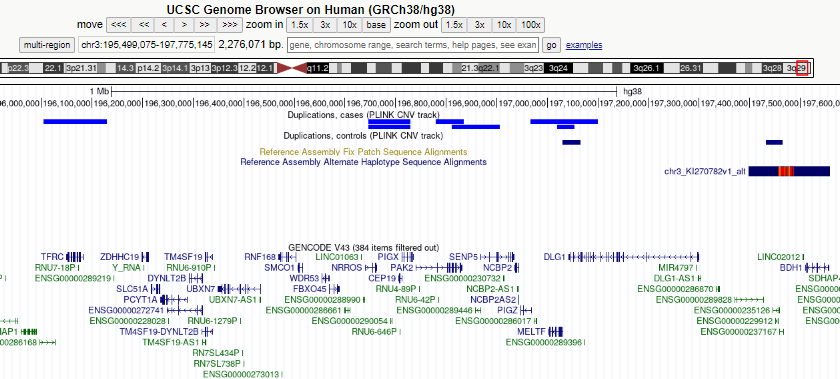


**Figure S7:** UCSC genome browser capture of ANGI CNVs annotated to the DECIPHER 3q29 duplication syndrome defined in ***Table S4***.

**Pleiotropic disease-risk dosage-sensitive CNVR associations**

Similar to within ANGI, we expanded testing for association with AN to 178 CNVRs reported to play a pleiotropic role in the development of 54 complex and Mendelian traits/disorders including 24 neurodevelopmental trait(9). To validate CNV annotation, control frequency comparison between UKB and ANGI revealed no difference (P > 0.05/178) for 170 out of 178 loci (96%) (Table S6). Out of the 178 CNVRs, 10 (6%) CNVRs had no UKB observations. No CNVRs showed significant AN-risk at the significance threshold corrected for multiple testing, but 7 were nominally significant (P < 0.05) after meta-analysis, including deletions within 2p22.3, and duplications within 12p13.33, 15q26.3, 3q29, 8p23.1, 2p22.3, 22q11.21 and 7q11.23 (***Table S5***).

The most significant association with AN was for duplications within cytoband 12p13.33 (12:190-1660kb) (P: 8.4x10-4), with an OR: 4.0 (CI: 0.91-38, P: 0.068) in ANGI and OR: 4.78 (CI: 1.59-10.9, P:8.8 x10-3) in the UKB (***Table S5).*** Encompassing ~1.5Mb and 10 genes, this region is linked to abnormalities in head, neck, and face(9). The average ANGI and UKB CNV annotated to the region are ~249kb and ~305kb, respectively. The control frequencies of the CNV were similar in the two datasets (UKB = 0.07%, ANGI = 0.02%, P = 0.27). Of the 12p13.33 duplications in 11 ANGI individuals (10 cases), 6 cases exhibited CNVs partially or fully duplicating B4GALNT3 (***Figure S8***), encoding GM2/GD2 synthase linked to neurodegenerative disorders(15). Moreover, CNVs intersecting genes SLC6A12 and SLC6A13, involved in GABA transport(15), were identified in 3 ANGI AN cases.

Of the 7 significant CNVRs four (3q29, 8p23.1, 22q11.2 and 7q11.23 duplications) represent cytobands with CNVs tested in the 67 syndromic set. However, the CNVRs differ in their start/stop base pair positions which impacts whether a called CNV is annotated to these regions (because annotation rules include percentage overlap).


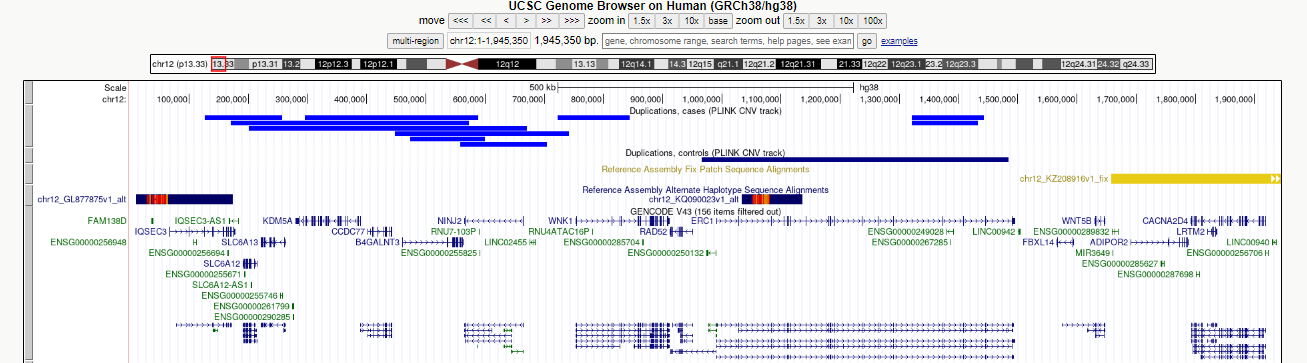


**Figure S8:** UCSC genome browser capture of ANGI duplications annotated to the pleiotropic, dosage-sensitive 12p13.33 duplication CNVR defined in ***Table S5.***

**Rare CNV breakpoint GWAS**

None of the novel AN-risk CNVRs identified within ANGI via a CNV-GWAS replicated a significant risk-effect in the UKB, although 6 (29%) CNVRs obtained P < 0.15 and were with AN risk (expect 7.5%), including a 63kb 16q21 deletion, an 80kb 5p12 deletion, a 388kb 7p22.1 (C1GALT1) duplication, a 100kb 7q31.1 (IMMP2L) deletion, and a 132kb 17q12 duplication (***Table S6***).

**Supplementary results: Syndromic CNV associations with BMI in ANGI and the UKB**

We compared the impact of the 67 syndromic loci on BMI in individuals with AN cases (**Table S7**) and separately in AN controls (**Table S8**). To help interpret the results, we note the lower age and lower BMI interquartile range in ANGI (lowest BMI) compared to the UKB (BMI at baseline visit) (**Table S2**). Following meta-analysis, no significant impact on BMI was found within AN cases for any of the 67 loci, and we replicate the established null effect of 3q29 duplications on BMI(16, 17). However, within the control group, 10 strong associations emerged driven by the UKB cohort. Considering only the UKB, we replicate the previously reported risk effect of 22q11 duplications on increased BMI (β: 0.63 kg/$m^{2}$, CI: 0.08-1.18, P:0.023). While 22q11 duplications are linked to obesity(16), there is no reported evidence for a “mirrored” BMI phenotype due to reciprocal 22q11 CNVs(16, 17). Our results are consistent with those reports, with no indication that 22q11 deletions are associated with a BMI reduction in cases or controls. Next, we replicate the known effects of the ~600kb 16p11.2 deletion, showing it is a potent driver of increased BMI in UKB controls (β: 5.5 kg/$m^{2}$, CI: 4.7-6.3, P: 7.5x${10}^{-46}$). Similarly, the parallel effect of the 220kb deletions was confirmed in the UKB controls (β: 4.1 kg/$m^{2}$, CI: 2.9-5.3, P: 6.8 x ${10}^{-11}$). In support of the 16p11.2 “mirror” hypothesis, we found duplications are significantly associated with reduced BMI for both the 220kb duplication (β: -1.3 kg/$m^{2}$, CI: -2.14 – 0.46, P: 0.0019) and ~600kb duplication (β: -1.6 kg/$m^{2}$, CI: -2.35-0.85, P: 3.4 x ${10}^{-5}$) in UKB controls.

**References**

1. Watson HJ, Yilmaz Z, Thornton LM, Hubel C, Coleman JRI, Gaspar HA, et al. Genome-wide association study identifies eight risk loci and implicates metabo-psychiatric origins for anorexia nervosa. Nat Genet. 2019;51(8):1207-14.

2. Thornton LM, Munn-Chernoff MA, Baker JH, Jureus A, Parker R, Henders AK, et al. The Anorexia Nervosa Genetics Initiative (ANGI): Overview and methods. Contemp Clin Trials. 2018;74:61-9.

3. Zhang Z, Cheng H, Hong X, Di Narzo AF, Franzen O, Peng S, et al. EnsembleCNV: an ensemble machine learning algorithm to identify and genotype copy number variation using SNP array data. Nucleic Acids Res. 2019;47(7):e39.

4. Pinto D, Pagnamenta AT, Klei L, Anney R, Merico D, Regan R, et al. Functional impact of global rare copy number variation in autism spectrum disorders. Nature. 2010;466(7304):368-72.

5. Wang K, Li M, Hadley D, Liu R, Glessner J, Grant SF, et al. PennCNV: an integrated hidden Markov model designed for high-resolution copy number variation detection in whole-genome SNP genotyping data. Genome Res. 2007;17(11):1665-74.

6. Colella S, Yau C, Taylor JM, Mirza G, Butler H, Clouston P, et al. QuantiSNP: an Objective Bayes Hidden-Markov Model to detect and accurately map copy number variation using SNP genotyping data. Nucleic Acids Res. 2007;35(6):2013-25.

7. Kent WJ, Sugnet CW, Furey TS, Roskin KM, Pringle TH, Zahler AM, et al. The human genome browser at UCSC. Genome Res. 2002;12(6):996-1006.

8. Kendall KM, Bracher-Smith M, Fitzpatrick H, Lynham A, Rees E, Escott-Price V, et al. Cognitive performance and functional outcomes of carriers of pathogenic copy number variants: analysis of the UK Biobank. Br J Psychiatry. 2019;214(5):297-304.

9. Collins RL, Glessner JT, Porcu E, Lepamets M, Brandon R, Lauricella C, et al. A cross-disorder dosage sensitivity map of the human genome. Cell. 2022;185(16):3041-55 e25.

10. Lisi EC, Hamosh A, Doheny KF, Squibb E, Jackson B, Galczynski R, et al. 3q29 interstitial microduplication: a new syndrome in a three-generation family. Am J Med Genet A. 2008;146A(5):601-9.

11. Vinas-Jornet M, Esteba-Castillo S, Baena N, Ribas-Vidal N, Ruiz A, Torrents-Rodas D, et al. High Incidence of Copy Number Variants in Adults with Intellectual Disability and Co-morbid Psychiatric Disorders. Behav Genet. 2018;48(4):323-36.

12. Pollak RM, Zinsmeister MC, Murphy MM, Zwick ME, Emory 3q P, Mulle JG. New phenotypes associated with 3q29 duplication syndrome: Results from the 3q29 registry. Am J Med Genet A. 2020;182(5):1152-66.

13. Green EK, Rees E, Walters JT, Smith KG, Forty L, Grozeva D, et al. Copy number variation in bipolar disorder. Mol Psychiatry. 2016;21(1):89-93.

14. Marshall CR, Howrigan DP, Merico D, Thiruvahindrapuram B, Wu W, Greer DS, et al. Contribution of copy number variants to schizophrenia from a genome-wide study of 41,321 subjects. Nat Genet. 2017;49(1):27-35.

15. Harlalka GV, Lehman A, Chioza B, Baple EL, Maroofian R, Cross H, et al. Mutations in B4GALNT1 (GM2 synthase) underlie a new disorder of ganglioside biosynthesis. Brain. 2013;136(Pt 12):3618-24.

16. Owen D, Bracher-Smith M, Kendall KM, Rees E, Einon M, Escott-Price V, et al. Effects of pathogenic CNVs on physical traits in participants of the UK Biobank. BMC Genomics. 2018;19(1):867.

17. Mace A, Tuke MA, Deelen P, Kristiansson K, Mattsson H, Noukas M, et al. CNV-association meta-analysis in 191,161 European adults reveals new loci associated with anthropometric traits. Nat Commun. 2017;8(1):744.
